# Design and performance of a small bath cryostat with NMR capability for transport of hyperpolarized samples

**DOI:** 10.1038/s41598-022-23890-7

**Published:** 2022-11-10

**Authors:** Andrea Capozzi

**Affiliations:** 1 SB IPHYS LIFMET, Institute of Physics, EPFL, CH F0 632, Bâtiment CH, Station 6, CH-1015 Lausanne, Switzerland; 2 Department of Health Technology, HYPERMAG, Technical University of Denmark, Building 349, 2800 Kgs Lyngby, Denmark

**Keywords:** Applied physics, Cancer screening

## Abstract

As of today, dissolution Dynamic Nuclear Polarization (dDNP) is the only clinically available hyperpolarization technique for ^13^C-MRI. Despite the clear path towards personalized medicine that dDNP is paving as an alternative and/or complement to Positron Emission Tomography (PET), the technique struggles to enter everyday clinical practice. Because of the minute-long hyperpolarization lifetime after dissolution, one of the reasons lies in the need and consequent complexities of having the machine that generates the hyperpolarization (i.e. the dDNP polarizer) on site. Since some years, research groups are working to make hyperpolarization transportable. Two different methods have been developed that allow “freezing” of the nuclear spin state prior to samples extraction from the polarizer. Nevertheless, so far, all attempts of transport have been limited to a very small scale and to the level of proof-of-principle experiments. The main reason for that is the lack of adequate hardware, strategy, and control on most of the crucial parameters. To bridge the technical gap with PET and provide MRI facilities with hours long relaxing hyperpolarized compounds at controlled conditions, a new generation of low cost/small footprint liquid He cryostats equipped with a magnetically enforced cryogenic probe is needed. In this paper, we detail the theoretical and practical construction of a hyperpolarized samples transportation device small enough to fit in a car and able to hold a sample at 4.2 K for almost 8 h despite the presence of a cryogenically-demanding purpose-built probe that provides enough magnetic field upon insertion of the sample and NMR quality homogeneity at storage position. Should transportable hyperpolarization via DNP become a reality, we herein provide important details to make it possible.

## Introduction

Invented in 2003, dissolution Dynamic Nuclear Polarization (dDNP)^1^ is undoubtedly the most versatile among the hyperpolarization techniques to increase NMR sensitivity in the liquid state. Its unmatched capability to investigate cellular metabolism in real time, by injecting hyperpolarized (HP) ^13^C-labelled molecules, has the potential to revolutionize diagnostic radiology enabling precision medicine and personalized healthcare^2–5^. Nevertheless, the methodology struggles to enter everyday clinical practice. One of the reasons why broad consensus among clinicians is still missing lies in the technical complexity that characterize hyperpolarization via dDNP^6–8^. A main drawback is that differently from PET, the current benchmark to investigate hypo and hyper metabolism in the clinic^9^, HP molecules prepared employing traditional polarizing agents cannot be transported far away from the production site without losing their high spin order and thus their increased NMR sensitivity^10^. Currently, should you be willing to equip an MRI facility with hyperpolarization, the only way is to place on site costly and technically demanding hardware (i.e. the dDNP polarizer). Lifting this restriction, making hyperpolarization transportable and disconnecting the production site of the HP molecules from the site of use, would represent a significant step forward.

Making hyperpolarization transportable entails, at the moment of extraction of the HP sample from the dDNP polarizer, the drastic reduction or elimination of the nuclear spins relaxation due to the same paramagnetic polarizing agents used, in the first place, to create the hyperpolarized state upon microwave irradiation^11,12^. So far, two approaches have been pursued:

1. To use UV-induced non-persistent radicals that can be quenched, after the DNP process has happened in the solid state, by heating up the sample above 190–200 K^13–17^;
2. To physically separate carbon nuclei from the polarizing agents by playing with radicals and ^13^C-labelled molecules with different solubility, or by grafting the radicals inside purpose synthesized porous polymers that can absorb a solution containing the substrate of interest^18–20^.

These two promising techniques are fundamentally different, but they share a common feature: after extraction from the polarizer, liquid helium (He) temperature and a magnetic field in the tesla range are required to store and transport a HP solid sample at conditions that guarantee a hours long relaxation time of the carbon nuclei^21,22^.

One could use a liquid He Dewar supplied by the local cryogenics’ provider and equip it with a permanent magnet^18^. Nevertheless, these Dewars are usually of large dimensions (60–100 L), they require special transportation, and have limited access to the He vessel from the top. The latter would compromise the possibility to employ a permanent magnet with the required magnetic field value and homogeneity to obtain a sufficiently long relaxation time of the HP sample and investigate its state with NMR, respectively. Using liquid nitrogen (N_2_) would simplify by far the cryogenics requirements, but would limit the relaxation time to few tens of minutes^14,21^.

Should transportable hyperpolarization become a reality, the aim of this paper is to provide the MR community with all key elements to make it possible. Namely, what would be needed to bridge the technical gap with delivered PET tracers is an easy-to-handle transportation device small enough to fit in a car, able to hold liquid He for hours, and equipped with a permanent magnets enforced NMR probe to, at the same time, shelter and measure the hyperpolarization state of the sample.

The design of small dimension cryostats is not a trivial task. The problem involves radiative, convective and, most of all conductive, heat transfer. Although it is not amenable to analytic solutions^23^, it is still possible to estimate numerically the feasibility of a design for some given dimensions and experimental constraints.

In this paper, inspired by the work from Caplin et al.^23^, we report an easy to implement numerical technique that helped us to assess the feasibility and to optimize the design of a non-conventional compact liquid He cryostat equipped with an intrinsically high thermal conductivity probe for the insertion, storage and NMR measurement of HP samples. We provide all details about the materials and construction of the cryostat, we analyse in depth the cryogenic performance, and we use NMR to characterize the magnetic field behaviour as a function of time and temperature of the storage magnet.

## Materials and methods

### Storage magnet and cryogenic probe

**Figure 1:**
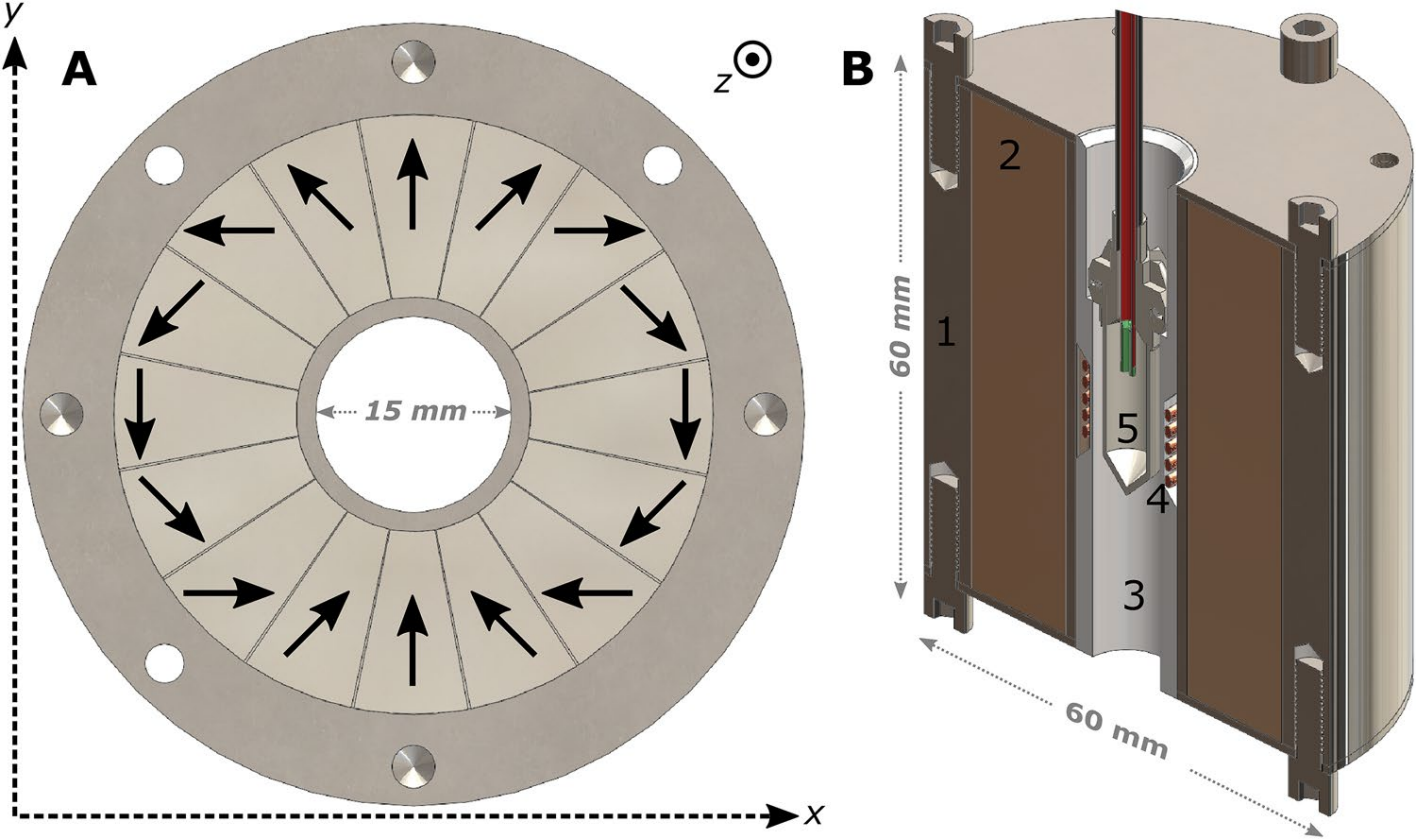
Drawings of the 16 elements Halbach magnet array. The arrangement and orientation of the different magnetic units is reported; the arrows represent the magnetization axis of the different elements (**A**). A simplified section view of the probe bottom part shows the sample position inside the Halbach magnet. The most important elements are indicated with numbers: stainless steel chassis of the Halbach magnet (1); SmCo magnetic material (2); PTFE coil former (3); solenoidal NMR coil (4); vial/sample cup (5).

The cryogenic probe of the transport device is home-built around a cylindrical Halbach magnet array (BuntingMagnetics Europe Ltd, Berkhamsted, Hertfordshire, England). The latter (Fig. 1) was custom designed in collaboration with and produced by BuntingMagnetics to fulfil the needs of a small footprint, a room temperature magnetic field value close to 1 T with NMR quality homogeneity in the absence of a shimming system, and minimal decrease of the residual magnetism (B_r_) at cryogenic temperature. The magnetic material is SmCo of grade YXG32, because of the smaller distortion of the magnetic domains, compared to the ubiquitous NdFeB, below 100 K ^24^. To improve field homogeneity, the magnetic ring is divided in 16 circular sectors of 22.5° each and magnetized in different directions in steps of 45°, to generate a field along the *y-axis* (Fig. 1A). Including the stainless-steel chassis (Fig. 1B, component 1), the magnet is a 62 mm tall hollow cylinder with a 60 mm OD and 15 mm ID. The bare magnetic material (Fig. 1B, component 2) is a 60 mm tall hollow cylinder with a 46 mm OD and 18 mm ID. A purpose machined PTFE coil former (Fig. 1B, component 3) is placed concentrically inside the magnet bore to align the vial/sample cup (i.e. bottom part of what we call a “Custom Fluid Path”^14^) to the isocenter of the magnet (Fig. 1B, component 5). With a declared homogeneity of at least 2000 ppm over a volume of 1 mL, a 5 turns solenoidal coil, parallel to the *z-axis*, and with a diameter and a height of 1 cm (Fig. 1B, component 4) is wrapped around the centre of the coil former.

The chassis of the Halbach array presents 3 × 3.5 mm pass through holes to allow sensors and cables to reach the bottom of the magnet, and 8 × M4 threading to mount the magnet on the cryogenic probe.

**Figure 2:**
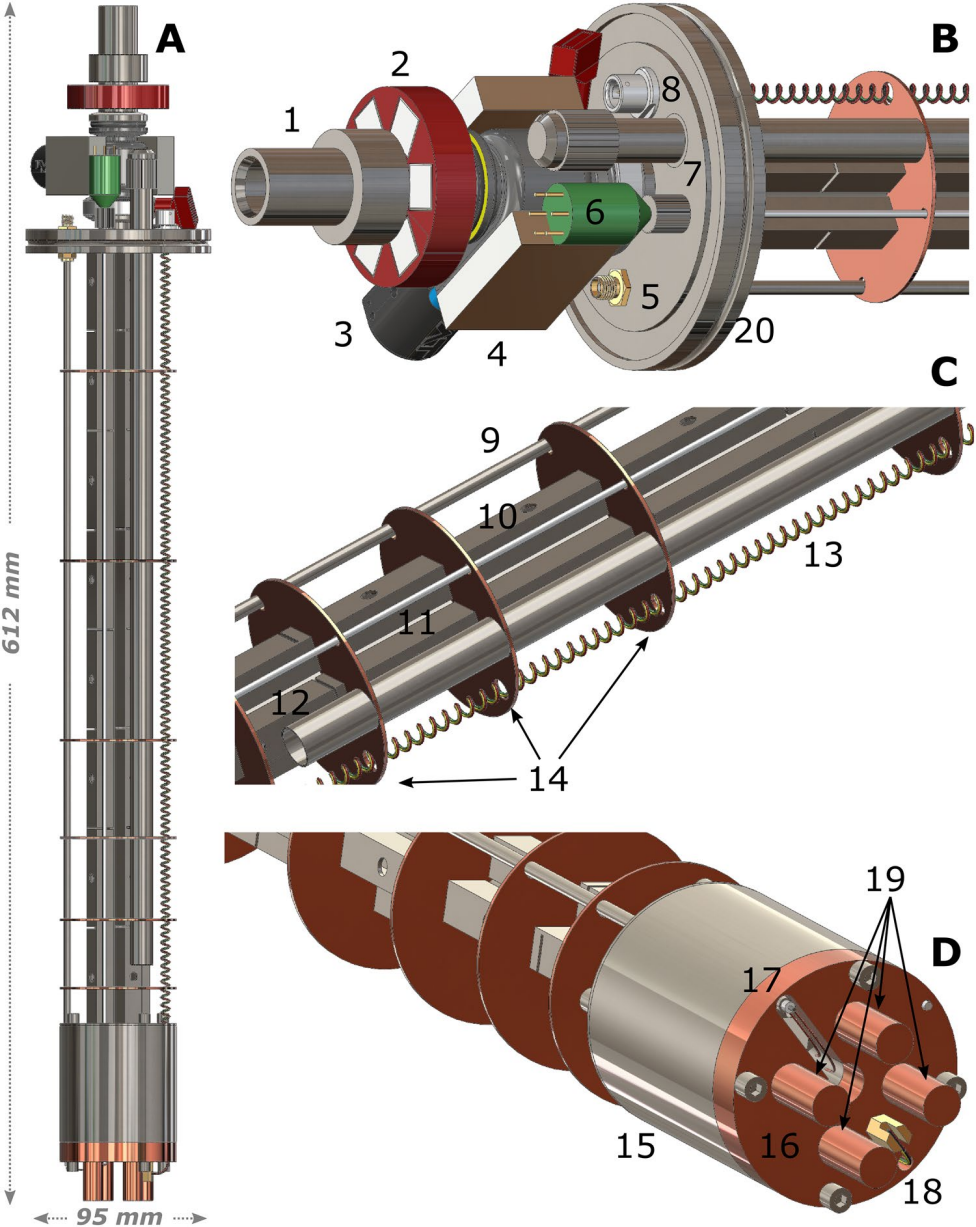
3D drawings of the probe built to transfer HP samples from the dDNP polarizer to the compact bath cryostat and store them inside a magnetic field and at a temperature of 4.2 K. We report its front view (**A**), as well as magnified pictures of the top part (**B**), middle part (**C**) and bottom part (**D**). The most important elements are indicated with numbers: loading chamber (1); six elements Halbach magnetic array around loading chamber (2); gate valve (3); two elements Halbach magnetic array around gate valve (4); NMR circuit SMA connector (5); liquid He probe pin connector (6); liquid He syphon port (7); Fischer connector for thermometer (8); NMR circuit coax cable (9); squared profile for four elements Halbach magnet array (10); liquid He probe stem (11); liquid He syphon (12); quartet of twisted cryogenic wires (13); copper horizontal radiation shields/baffles (14); sixteen elements storage Halbach magnetic array (15); copper bottom (16); coax cable termination with connection to the solenoidal coil (17); cernox temperature sensor (18); probe’s copper tails (19).

The cryogenic NMR probe (Fig. 2) is conceptually similar, but more compact in size, to the one presented in a former study to extract the HP samples from the dDNP polarizer, while retaining the high spin order^14^. Here, in a similar way, we provide enough magnetic field (i.e. 40 mT at least)^14,18^, from the top of the probe to the storage magnet (Fig. 2A) by using off-the-shelf NdFeB permanent magnets (Supermagnete, Gottmadingen, Germany).

The sample loading chamber (Fig. 2B, component 1), used to move the HP extracted sample from the dDNP polarizer to the transport device^14^, is surrounded by a 3D-printed structure (MK3S, Prusa Research a.s., Prague, Czech Republic) that holds in place a six elements Halbach array (12 mm × 12 mm × 12 mm block magnets; Fig. 2B, component 2). The mini gate valve (VAT Vakuumventile AG, Switzerland; Fig. 2B, component 3) is in between a two elements Halbach array (46 mm × 30 mm × 10 mm block magnets; Fig. 2B, component 4) glued to its surfaces. The 13 mm OD/12 mm ID stainless steel sample loading tube (Interalloy AG, Schinznach-Bad, Switzerland; not clearly visible in Fig. 2) is enclosed inside a four elements Halbach array (7.6 mm × 7.6 mm × 50 mm block magnets) held in place by stainless-steel squared profiles of 10 mm × 10 mm OS/8 mm × 8 mm IS (Interalloy AG, Schinznach-Bad, Switzerland, Fig. 2B, component 10). This Halbach arrangement is repeated 8 times from the top to the bottom of the sample loading tube. To reduce thermal conductivity, each magnet block is separated by the following one on the *z-axis* using a 1 mm thick PTFE disc (not shown) and, in correspondence of the disc, the stainless-steel squared profile is cut on 3 sides out of 4. The squared profiles also have threaded holes on one face to help assembling the magnetic elements.

To cover the loading chamber/mini gate valve and mini gate valve/top flange gaps, the ISO-KF-16 half nipples of the loading chamber and top flange (Fig. 2B, component 1) are filled with 8 elements Halbach arrays (1.5 mm × 1 mm × 5 mm block magnets) over a length of 20 mm^14^. It is important to notice that all magnetic Halbach arrays are mounted such as to generate a field aligned with the one of the storage magnet along the *y-axis* (Fig. 1).

Along the length of the probe, six copper baffles (Fig. 2C, component 14) are silver soldered to the stainless-steel body at uneven distances. The 3 lower ones are closer to each other and are meant to cut convection modes inside the cryostat. The 3 upper ones are placed in correspondence of the position of the two radiation shields and the top flange of the cryostat (see paragraph 2.2 and 2.3) and are meant to cut heat conduction via radiation and to dump any temperature gradient in the x–y plane. The last baffle at the bottom is made of stainless-steel (not clearly visible in Fig. 2) and allows to attach the storage magnet (Fig. 2D, component 15) to the probe body. A 10 mm thick copper plate (Fig. 2D, component 16) with four cylindrical copper tails (Fig. 2D, component 19) is screwed at the bottom of the magnet to protect the Cernox temperature sensor (CX-1050-MT-1.4L-QL, Lake Shore Cryotronics, Westerville, USA; Fig. 2B, component 18) and always ensure a good thermal contact between the magnet and the liquid He, especially when the liquid He level is low. Four cryogenic wires (Quad-Lead™, Lake Shore Cryotronics, Westerville, USA; Fig. 2C, component 13) connect the temperature sensor to a feedthrough 4 pin connector (WDE 102 AZ053-130, Fischer Connectors SA, Saint-Prex, Switzerland; Fig. 2B, component 8). A 500 mm long liquid He probe with a sensitive length of 150 mm (HLP-AL150-TL500, Cryogenic Ltd, London, England) runs from the top flange (Fig. 2B, component 6), through all baffles (Fig. 2C, component 11), to the bottom of the copper plate (zero level for the liquid He measurements). The solenoidal coil placed inside the custom magnet, is soldered (Fig. 2D, component 17) to a reduced thermal conductivity semi-rigid coax cable (0.141SS-W-P-50, Jyebao, Taiwan; Fig. 2C, component 9) that connects it to a feedthrough SMA (SF-2991-6002, Amphenol SV Microwave, West Palm Beach, USA; Fig. 2B, component 5) to the top flange. Finally, a 400 mm long/11 mm OD/10.5 mm ID stainless-steel tube acts as syphon (Fig. 2C, component 12) and allows to top-up liquid He, even in presence of a sample, from the filling port placed on the top flange (Fig. 2B, component 7). The most compact solution to fit all these elements at the top is an ISO-K-63 blank flange (Pfeiffer Vacuum, Asslar, Germany; Fig. 2B, component 20).

### Thermodynamic model for compact cryostat design

**Figure 3:**
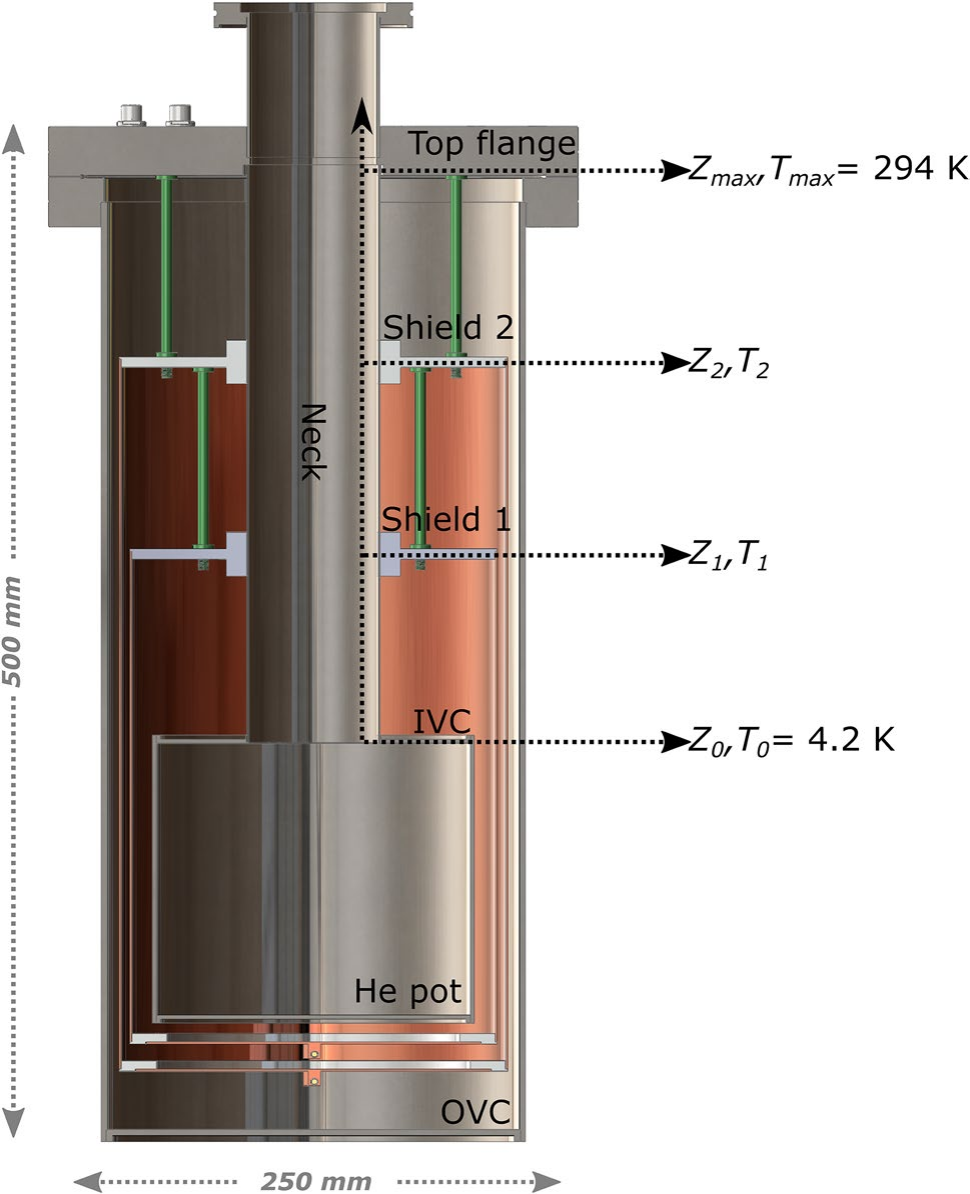
Reference sketch for the numerical model of the cryostat. From top to bottom we find the *top flange* placed at height *Z*_*max*_ and room temperature (294 K); the *shield 2* placed at height *Z*_*2*_ and temperature *T*_*2*_; the *shield 1* placed at height *Z*_*1*_ < *Z*_*2*_ and *T*_*1*_ < *T*_*2*_; the *He pot* whose top part is placed at *Z*_*0*_ and its temperature is considered fixed at *T*_*0*_ = 4.2 K, despite the liquid He level. The *top flange* together with the *neck* and the *He pot* form the *IVC* (inner vacuum chamber). The dotted horizontal black arrows indicate position and temperature of the elements listed above. The dotted vertical black arrow indicates the direction for increasing value of the *z-coordinate*. The *OVC* (outer vacuum chamber) closes the cryostat and the vacuum in between *IVC* and *OVC* very good (e.g., 10^–8^–10^–7^ mbar). *IVC* and *OVC* are made of stainless-steel, while *shield 1* and *shield 2* are made of copper. Nevertheless, for IVC, shield 1 and shield 2 we consider the aluminium emissivity because they are wrapped with MLI (multi-insulation layers). The green rods represent polyamide-imide spacers to support the radiation shields.

Willing to build a compact and transportable device (refer to Fig. 3 for all elements recalled in this paragraph), we consider a bucket cryostat with a non-uniform cross section of the inner vacuum chamber (IVC). This allows to increase the amount of liquid He that can be condensed while keeping the total height limited (outer vacuum chamber, OVC, close to 500 mm and IVC ≤ 400 mm). We assume that the cryogenic probe (not represented in Fig. 3 for simplicity) placed inside the neck of the cryostat has enough baffles to cut any convection of the He gas and smooth out any horizontal temperature gradient. Therefore, we can expect that^23,25^:

1. There are no heat sources from the outside other than radiation and conduction.
2. The thermal contact between the cold cryogenic gas, that flows inside the neck, and its surroundings is good enough to neglect all temperature variations across any horizontal cross section, simplifying the problem to a mono-dimensional one.
3. The direct input from room temperature radiation shining straight down the cryostat can be neglected because of the presence of the baffles.

When designing a cryostat, the fundamental physics question is to determine the best temperature profile from the He pot (surface at 4.2 K) to the top flange (surface at room temperature, 294 K) that minimizes the liquid He consumption. As a rule of thumb, the steeper is the profile, the “faster” is the gas flow needed to maintain it, thus the He consumption. For a closed system at constant pressure, as is a cryostat, when the steady state is achieved, the “cooling power” of the cryogenic gas must equal at any height $z$, where the temperature is $T\left( z \right)$, the heat input $\delta Q$ from above. Formally, the “cooling power” can be expressed as the gas enthalpy rate $dH$, i.e. the amount of “cold” gas at temperature $T\left( z \right)$ crossing the planar surface placed at $z$ every second. Therefore, for each cross-section of the cryostat neck we can write the Equation^23^:

1 $${\delta Q} = {\text{dH}}\left( {\text{T}} \right) = {\mu H}\left( {\text{T}} \right) = {\mu c}_{{\text{p}}} {\text{T}}$$

where $\mu$ is the He gas flow rate in $mol/s$ and $H\left( T \right)$ is the He gas molar enthalpy with $c_{P} = 5/2R = 20.7 J \cdot mol^{ - 1} \cdot K^{ - 1}$ being the specific heat at constant pressure for a mono-atomic gas. Here, for simplicity we don’t consider the latent heat of evaporation of He because it is small enough to be neglected^23^.

As anticipated earlier, the first contribution to the heat input $\delta Q$ is the conduction. At the steady state we can express it by the time independent Fourier’s law:

2 $${\delta Q}_{1} = - {\text{K}}\left( {\text{T}} \right)\frac{{{\text{dT}}}}{{{\text{dz}}}}$$

In Eq. (2), $K\left( T \right)$ is the temperature dependent conductance that is the sum of two terms: all stainless steel parts of the cryostat $CS_{steel} k_{steel} \left( T \right)$, with $CS_{steel}$ the total cross section (cryostat neck, probe loading tube, probe four squared profiles, probe He syphon, coax cable, liquid He probe) and $k_{steel} \left( T \right)$ the temperature dependent conductivity of stainless-steel 304L; the He gas column itself $CS_{He} k_{He} \left( T \right)$, with $CS_{He}$ the cross section of the He gas column and $k_{He} \left( T \right)$ the temperature dependent conductivity of He gas. $\frac{dT}{{dz}}$ is the temperature gradient along the neck.

The second contribution to the heat input $\delta Q$ is the external radiation reaching the He pot that can be expressed by the Stefan-Boltzmann law as:

3 $${\delta Q}_{2} = {\upsigma }_{{{\text{SB}}}} {\text{rad}}_{{{\text{OVC}} \to {\text{He pot}}}} \left( {{\text{T}}_{{{\text{OVC}}}}^{4} - {\text{T}}_{{\text{He pot}}}^{4} } \right)$$

In Eq. (3), $\sigma_{SB} = 5.67e^{ - 8} W \cdot m^{ - 2} \cdot K^{ - 4}$ is the Stefan-Boltzmann constant; $rad_{{{\text{OVC}} \to {\text{He pot}}}}$ is the fraction of radiation leaving the cryostat external surface and reaching the He pot; $T_{{{\text{OVC}}}}$ and $T_{He pot}$ are the temperature of the two concerned surfaces, respectively.

In He cryostat design, to reduce the impact of radiation on the He pot, it is usual practice to “break” this contribution by adding radiation shields (cylindrical metal surfaces at a given temperature anchored to the cryostat neck).

Therefore, we can generalize Eq. (3) for any pair of surfaces placed at $Z_{n} < Z_{n + 1}$ with temperatures $T_{n} < T_{n + 1}$ as:

4 $${\delta Q}_{{2{\text{n}}}} = {\upsigma }_{{{\text{SB}}}} {\text{rad}}_{{{\text{n}} + 1 \to {\text{n}}}} \left( {{\text{T}}_{{{\text{n}} + 1}}^{4} - {\text{T}}_{{\text{n}}}^{4} } \right)$$

where $rad_{n + 1 \to n} = \frac{{S_{n} VF_{n + 1 \to n} }}{{\frac{1}{{\varepsilon_{n} }} + \frac{{S_{n} }}{{S_{n + 1} }}\left( {\frac{1}{{\varepsilon_{n + 1} }} - 1} \right)}}$ is the fraction of radiation emitted by the cylindrical surface anchored to the cryostat neck at $Z_{n + 1}$ and absorbed by the cylindrical surface anchored to the cryostat neck at $Z_{n}$, with $S_{n,n + 1} and \varepsilon_{n,n + 1}$ the surface and emissivity of the $n,n + 1$ cylinder, and $VF_{n + 1 \to n}$ the geometrical view factor between the two (see Supporting Information).

Putting all together, for a cross-section at height $z$, with $Z_{n} < z < Z_{n + 1}$ Eq. (1) can be rewritten as:

5 $${\delta Q} = {\delta Q}_{1} + {\delta Q}_{{2{\text{n}}}} = \left( {{\text{CS}}_{{{\text{steel}}}} {\text{k}}_{{{\text{steel}}}} \left( {\text{T}} \right) + {\text{CS}}_{{{\text{He}}}} {\text{k}}_{{{\text{He}}}} \left( {\text{T}} \right)} \right)\frac{{{\text{dT}}}}{{{\text{dz}}}} + {\upsigma }_{{{\text{SB}}}} {\text{rad}}_{{{\text{n}} + 1 \to {\text{n}}}} \left( {{\text{T}}_{{{\text{n}} + 1}}^{4} - {\text{T}}_{{\text{n}}}^{4} } \right) = {\mu c}_{{\text{p}}} {\text{T}}$$

For small cryostats, no more than 2 radiation shields are generally installed^25^. Following this recommendation, for a given set of He boil-off rate, length of the neck and position/temperature of the shields, to determine the temperature profile, Eq. (5) can be integrated in three steps from ${z= Z}_{0}$ to ${z= Z}_{1}$, from ${z= Z}_{1}$ to ${z= Z}_{2}$, and from ${z= Z}_{2}$ to ${Z=Z}_{max}$, with ${Z}_{0}$, ${Z}_{1}$, ${Z}_{2}$, ${Z}_{max}$ the position of He pot, shield 1, shield 2 and OVC, respectively. The boundary conditions are that the flow has the same value in all sections and that the end temperature of the *n*^*th*^-section is equal to the start temperature of the *n* + *1*^*th*^-section. At the beginning of each section, respectively ${Z}_{0}$, ${Z}_{1}$ and ${Z}_{2}$, the net radiation contribution acts as “an electric heater”, and it provides a heat input independent of *z-coordinate* until the beginning of the next section. It is important to notice that the conduction part of Eq. (5) appears with a positive sign because we integrate from “low temperature” to “high temperature”.

To integrate Eq. (5) we use the MATLAB (The MathWorks, Natick, Massachusetts, USA) ODE solver ode45 that requires the equation to be written in the form

6 $$\frac{{{\text{dT}}}}{{{\text{dz}}}} = \frac{{{\mu c}_{{\text{p}}} {\text{T}} - {\upsigma }_{{{\text{SB}}}} {\text{rad}}_{{{\text{n}} + 1 \to {\text{n}}}} \left( {{\text{T}}_{{{\text{n}} + 1}}^{4} - {\text{T}}_{{\text{n}}}^{4} } \right)}}{{{\text{CS}}_{{{\text{steel}}}} {\text{k}}_{{{\text{steel}}}} \left( {\text{T}} \right) + {\text{CS}}_{{{\text{He}}}} {\text{k}}_{{{\text{He}}}} \left( {\text{T}} \right)}}$$

The temperature dependent conductivity for stain-less steel 304L and for the He gas is obtained as polynomial fits of data tables^26,27^ (see Supporting Information, Figure S1). For simplicity, the magnetic structures around the sample loading tube of the probe are considered as continuous stainless steel square rods. Being the cross section of the four bar magnets 80% of ${CS}_{steel}$, to take them into account for the heat transfer computation, Eq. (6) rewrites:

7 $$\frac{{{\text{dT}}}}{{{\text{dz}}}} = \frac{{{\mu c}_{{\text{p}}} {\text{T}} - {\upsigma }_{{{\text{SB}}}} {\text{rad}}_{{{\text{n}} + 1 \to {\text{n}}}} \left( {{\text{T}}_{{{\text{n}} + 1}}^{4} - {\text{T}}_{{\text{n}}}^{4} } \right)}}{{1.8 \cdot {\text{CS}}_{{{\text{steel}}}} {\text{k}}_{{{\text{steel}}}} \left( {\text{T}} \right) + {\text{CS}}_{{{\text{He}}}} {\text{k}}_{{{\text{He}}}} \left( {\text{T}} \right)}}$$

As a rule of thumb, the two radiation shields are meant to split the temperature gradient evenly. Therefore, concerning the emissivity of the different surfaces of the cryostat, we consider the values at 4.2 K, 100 K, 200 K and 294 K for the He pot, shield 1, shield 2 and the OVC, respectively. The material emissivity is aluminum for the first 3 surfaces (wrapping of the concerned elements in a 4-layer MLI, Herose Ltd, Doncaster, GB), and stainless steel for the OVC.

Table 1 summarizes all important geometrical dimensions and value of the constants used in the model.

**Table 1:** All components, geometrical dimensions and parameters used in the model are listed here.

Conduction							
Component name	Component number	Component material	OD/OS (mm)	ID/IS (mm)	Cross sec. (mm^2^)	Conductivity 294 K W·(K·m)^-1^	Conductivity 4.2 K W·(K·m)^-1^
Loading tube	1	SS 304L	13.0	12.0	19.63	15	0.240
He level probe	1	SS 304L	2.0	0.0	3.14	15	0.240
Squared profile	4	SS 304L	10.0*	8.0*	36	15	0.240
Block magnets	4	NdFeB	7.6*	0	57.76	15**	0.240**
Coax cable	1	SS 304L	3.2	3.0	0.97	15	0.240
He syphon	1	SS 304L	11.5	10.5	17.27	15	0.240
Neck	1	SS 304L	63.5	62.5	98.69	15	0.240
Probe conduction part	13	SS 304L	\	\	514.74	15	0.240
He conduction part	1	He gas	62.5	0.0	3066.40	0.15	0.009
**Radiation**							
Component name	Component number	Component material	OD (mm)	ID (mm)	Height*** (mm)	Surface*** (mm^2^)	Emissivity
He pot	1	SS 304L	154	150	130	55·10^3^	0.04 (4.2 K)
Shield 1	1	Copper	175	173	235	128·10^3^	0.08 (100 K)
Shield 2	1	Copper	185	183	340	202·10^3^	0.1 (200 K)
OVC	1	SS 304L	201	197	450	290·10^3^	0.2 (294 K)

We report only the thermal conductivity of stainless steel (SS 304L) and He gas at 294 K and 4.2 K as an example; its temperature dependence is derived into the MATLAB script (see Supporting Information, Figure S1). For the squared profile OS and IS represent its external and internal dimension and not diameter (*). The NdFeB thermal conductivity is assumed identical to SS 304L (**). Concerning the height and thus the surfaces of the different components, we report the values chosen to build the cryostat (***).

### Cryostat construction details

**Figure 4:**
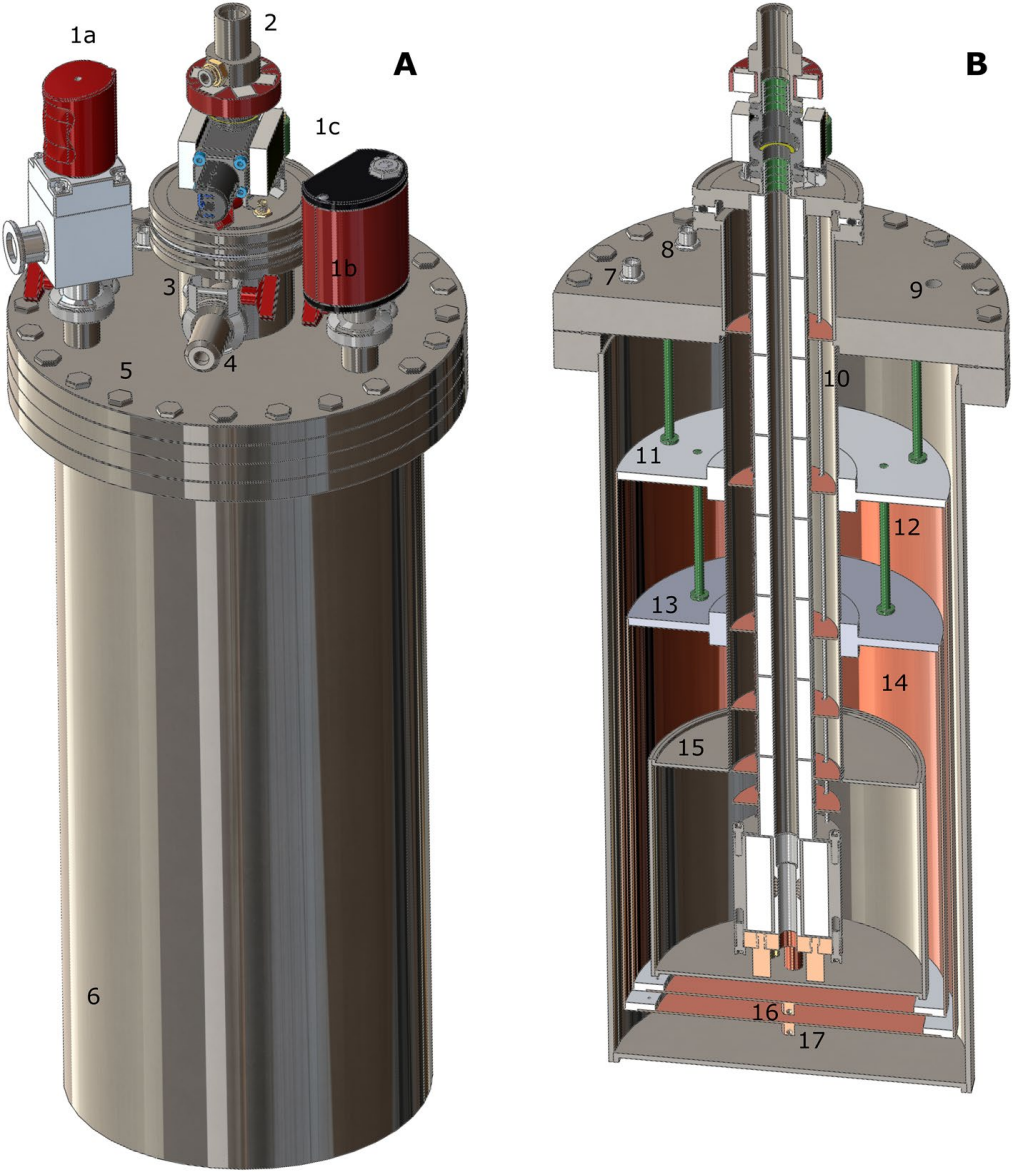
3D drawings (**A**) and section view (**B**) of the full cryostat with probe. OVC pumping valve (1a); pressure gauge (1b); OVC overpressure valve (1c, not visible); top of the probe (2); ISO-K-63 half-nipple (3); boil off check valve (4); CF-200 top flange (5); OVC (6); outer shield/shield 2 Fischer connector (7); inner shield/shield 1 Fischer connector (8); dissolution stick M10 threading (9); shields ‘mounting rods (10); outer shield/shield 2’s heat exchanger (11); outer shield/shield 2 (12); inner shield/shield 1’s heat exchanger (13); inner shield/shield 1 (14); He pot (15); inner shield/shield 1’s temperature sensor (16); outer shield/shield 2’s temperature sensor (17).

A 3D view and a coronal section view of the complete transportation device are reported in Fig. 4A,B, respectively. The cryostat is built around the ISO-K-63 flange of the probe (Fig. 4A, component 2). An ISO-K-63 half nipple (Pfeiffer Vacuum, Asslar, Germany; Fig. 4A, component 3) cut at 74 mm is welded to a CF-200 threaded blank flange (Pfeiffer Vacuum, Asslar, Germany; Fig. 4A, component 5) milled accordingly. The latter presents also three more ISO-KF-16 opening to accommodate a pumping port (Pfeiffer Vacuum, Asslar, Germany; Fig. 4A, component 1a), a pressure gauge (Pfeiffer Vacuum, Asslar, Germany; Fig. 4A, component 1b) and a safety valve (VAT Vakuumventile AG, Switzerland; Fig. 4A, component 1c not visible). The ISO-K-63 half-nipple presents an ISO-KF-16 opening on the cylindrical surface to connect a He boil-off check valve (Herose Ltd, Doncaster, GB; Fig. 4A, component 4). Finally, the CF-200 flange has two holes to lodge two feedthrough 4 pin connectors (WDE 102 AZ053-130, Fischer Connectors SA, Saint-Prex, Switzerland; Fig. 4B, components 7 and 8) and a M10 blind threading for mounting purposes (Fig. 4B, component 9). The OVC is sealed by compressing a copper gasket between the CF-200 flange and a 450 mm tall CF-200 threaded half-nipple (Pfeiffer Vacuum, Asslar, Germany; Fig. 4A, component 6). The IVC is completed by welding the top flange to the neck, a stainless-steel tube with 63.5 mm OD/62.5 mm ID (Interalloy AG, Schinznach-Bad, Switzerland; Fig. 4B, component 10), and the latter to the He pot, a stainless-steel tube with 154 mm OD/150 mm ID (Interalloy AG, Schinznach-Bad, Switzerland; Fig. 4B, component 15). The copper outer shield/shield 2 and inner shield/shield 1 (Fig. 4B, components 12 and 14) are connected to the neck by means of two aluminium heat exchangers (Fig. 4B, components 11 and 13). To insure good thermal contact, the heat exchangers squeeze the cryostat neck on a length of 20 mm by means of 2 transversal screws, one above the aluminium plate and one below. Moreover, their distance from the top flange and mechanical stability is provided by non-conductive polyamide-imide plastic supports (green rods in Fig. 4). The radiation shields are closed at the bottom with two copper lids, each of them silver soldered to a small copper cube (Fig. 4B, components 16 and 17) that accommodates a diode temperature sensor (DT-670-MT-1.4L-QL, Lake Shore Cryotronics, Westerville, USA). The temperature sensors are connected to the hermetic Fischer connectors on the CF-200 flange using cryogenic wires. The optimal length of the neck and the position of the shields were chosen according to the result of simulations (see *Results and Discussion*); all other dimensions are reported in Table 1. As mentioned before, the IVC and the shields are wrapped in MIL (not shown in Fig. 4).

### Cryostat routine operations and measurements

The three temperature sensors (Cernox for the magnet and diodes for the radiation shields) are connected to three different single channel temperature monitor devices (LS-211-S, Lake Shore Cryotronics, Westerville, USA), each of them uploaded with the characteristic calibration curve of the sensor. The liquid He probe is connected to a He level gauge console (HLG-200, Cryogenic Ltd, London, England). A home-made LabView (National Instruments, Austin, TX, USA) software monitors all three temperatures and the He level as a function of time and logs the data. This set up is used for measuring the performance of the cryostat in terms of cooling to 4.2 K, filling of the He pot, and holding of the cryogenic liquid.

Upon first use or after several months, the OVC is pumped overnight to reach a pressure of 10^–7^ mbar. The cooldown and filling procedure happens in three steps. Firstly, thanks to its higher enthalpy compared to liquid He, liquid N_2_ is used to cool down the cryostat from room temperature to 77 K. Secondly, as soon as the target temperature has been reached, the liquid N_2_ flow is stopped and the condensed cryogenic pushed out using He gas. Thirdly, when the temperature starts to increase above 77 K, liquid He is used for the last cooling step to 4.2 K and complete filling of the He pot. Finally, the liquid He dewar is disconnected and the cryostat is ready to accommodate a sample.

### Measurement of the storage magnet field drift

**Figure 5:**
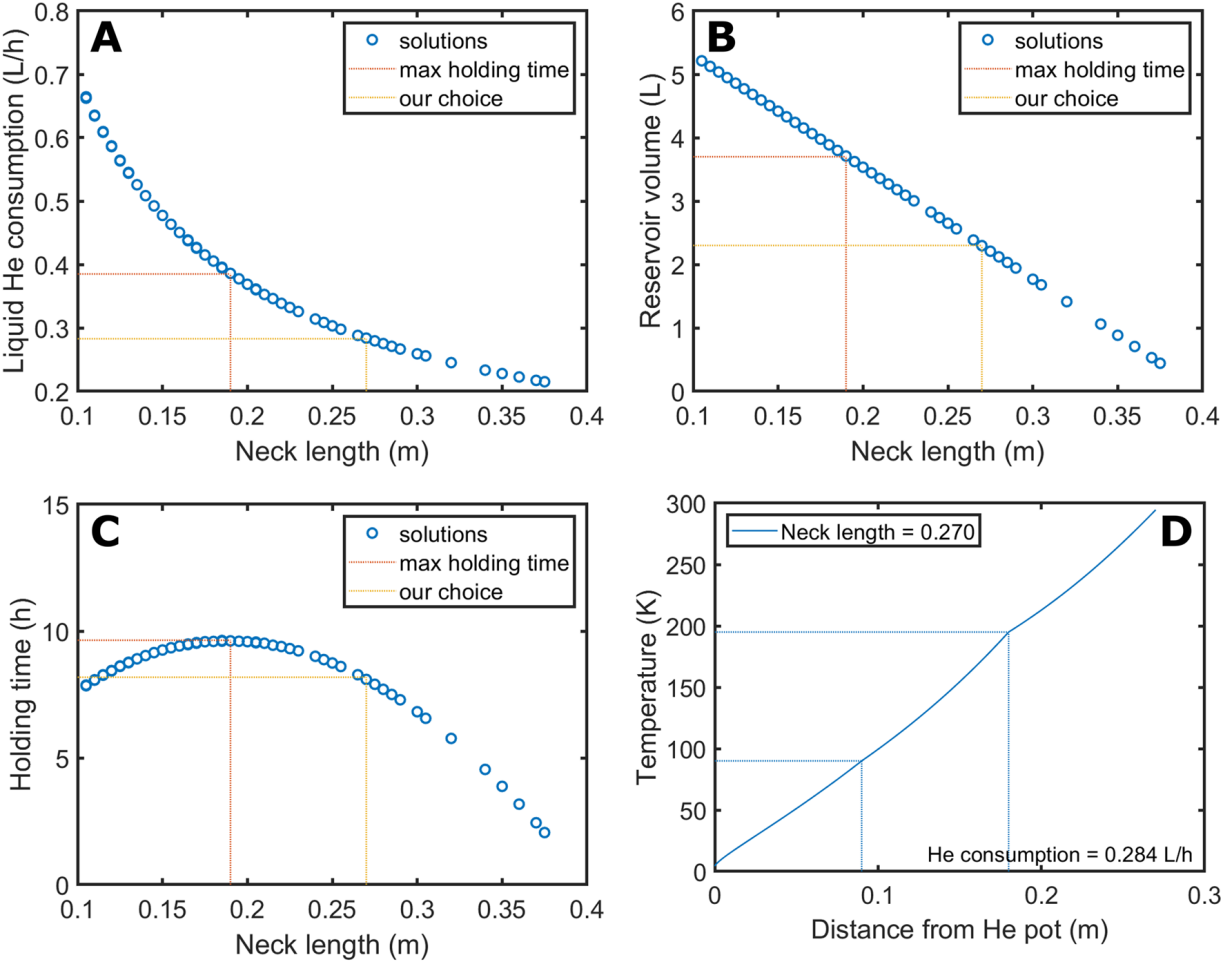
Liquid He consumption as a function of the cryostat neck length. The graph reports all gas flow value that solve Eq. (4), for a given length of the neck (**A**). All values of the neck length leading to a solution are used to calculate the consequent volume of the He pot (**B**). The ratio between He pot volume and consumption rate yields the maximum holding time as a function of the neck length (**C**). The orange and yellow dotted lines indicate the parameters corresponding to the maximum holding time and the choice we made to build the cryostat, respectively. The temperature profile of the configuration of choice without optimization of the positions of the radiation shields is reported in (**D**).

A 1.5 m long cryogenic Hall probe (LS-MCT-3160-WN, Lake Shore Cryotronics, Westerville, USA) connected to a gauss meter (LS-475-DSP, Lake Shore Cryotronics, Westerville, USA) is used in a single experiment to measure the storage magnet field drift as a function of the temperature. After filling the magnet with liquid He as described in paragraph 2.4, the Hall probe is slit inside the sample tube. The coaxial alignment of the Hall probe with the storage magnet bore and the positioning of its sensitive part inside the isocenter of the storage magnet are guaranteed by two 3D printed supports: the first being a ISO-KF-16 flanged cylinder with an ID making a tight fit with the Hall probe itself and mounted instead of the loading stem (Figs. 2B, 1); the second one being a “bottom free” sample vial (Figs. 1B, 5) mounted above the sensitive area of the Hall probe. A home-made LabView (National Instruments, Austin, TX, USA) software is employed to measure, once every 5 min, the value of the magnetic field and the temperature of the magnet. The experiment runs over 2 days until the magnet warms up to room temperature.

### ^1^H NMR measurements

The Hall probe provides a measurement of the magnetic field with a precision of 0.1 mT that corresponds to a NMR resolution in the kHz range. Starting from the values measured with the Hall probe at 4.2 K, 77 K and 294 K, a better estimation of the magnetic field is performed using ^1^H NMR on a 125 μL sample containing 50 mM TEMPOL radical dissolved in a glycerol:water 1:1 (v/v) solution. The three measurements are performed at “bath conditions” using liquid He, liquid N_2_ and an empty warm cryostat, respectively. The coil is remotely tuned and matched outside of the cryostat by means of a T/M box with two piston trimmer capacitors (Voltronics V1949) connected to the cryogenic probe through the SMA (Fig. 2B, 5)^28,29^. The T/M box can cover the range 38–42 MHz. The NMR signal is acquired at each temperature using a compact bench-top spectrometer (Kea2, Magritek, Wellington, New Zealand). A single 90° hard pulse is enough to get a good SNR. The NMR experiment is repeated 5 times every 2 days to evaluate the stability of the magnet across complete temperature cycles.

### Magnetic field simulations and data analysis

All NMR data are processed in MNOVA (Mestrelab Research, Santiago de Compostela, Spain). Magnetic field simulations are performed using COMSOL 5.4 (COMSOL Multiphysics, Burlington, Massachusetts, U.S.)^14^. Plots from measured data are generated using Origin 2019 (OriginLab Corporation, Northampton, Massachusetts, U.S.). Plots from simulations are generated using MATLAB (Mathworks, Natick, MA, U.S.). All 3D, 2D drawings of the cryostat are made using SOLIDWORKS 2021 (Dassault Systèmes, Vélizy-Villacoublay, France).

### Statistical analysis

Measurement of the cryostat holding time, liquid He consumption and temperature reached by the radiation shields were repeated several times across 1 year of use. A batch of 5 measurements collected in different months is considered for statistical analysis. The results in the main text are reported as average of repeated measurements, and the standard deviation represents the error. Although the cooldown procedure was monitored as many times as the holding time measurement, its execution was too much dependent on factors as the pressure value of the liquid N_2_ dewar and in the He recovery line of the lab. The latter prevented us from performing experiments at repeatable conditions. Therefore, in the results section we report a typical “cool down curve”, but no statistical analysis is performed in this case. The measurement of the shift of the magnetic field value as a function of the temperature was performed only once because it was used simply as starting point to find the ^1^H NMR resonance at 4.2 K, 77 K and 294 K.

## Results and discussion

### Compact cryostat feasibility and optimization of the construction

Thermal radiation is by far the most important contribution to the thermal budget in cryostats. When dealing with liquid He, the presence of radiation shields is crucial to reduce the impact on the cryogenic container and prolong or even make it possible to condense the gas^30^. Nevertheless, when the dimensions of the cryostat are small (e.g. height < 500 mm), thermal conduction can also play an important role. Moreover, in our case heat conduction is even more critical because of the presence of a bulky-permanent magnets enforced NMR probe.

Willing to assess the feasibility of the design, we firstly use the model to choose a suitable length of the cryostat neck that can guarantee a holding time of at least 8 h (one working day) while keeping the IVC height = 400 mm. In this case, following common practice in cryostats design^23,31^, we place the first and second radiation shields at a distance from the He pot equal to 1/3 and 2/3 of the neck length, respectively. The height of the He pot, and thus its volume, is variable and depends on the length of the neck. For a given set of value of the gas flow and position of the shields, the length of the neck is changed in steps of 5 mm from 105 to 395 mm. The condition of acceptance of the solution is on the resulting end temperature at the top flange ($T_{end}$): $\left| {T_{end} - 294} \right| < 0.5 K$. Figure 5A shows the values of the gas flow that solves Eq. (7) for different lengths of the cryostat neck.

To ease the interpretation of the data, we prefer to express the He gas flow as “consumption of liquid He”. To do so, each value in mol/s is multiplied by 22.4·1/3600 to obtain the gas flow in L/h and divided by 757 that is the liquid He expansion coefficient. As expected from Eq. (2), the liquid He consumption decreases by increasing the length of the cryostat neck (i.e. the integration interval on $z$), from a maximum of 0.663 L/h (neck length of 105 mm) to a minimum of 0.215 L/h (neck length of 375 mm). The volume of the He pot decreases linearly for increasing length of the neck from a maxim of 5.2 L to a minimum of 0.4 L (see Fig. 5B). Consequently, the maximum holding time (i.e. He pot volume/liquid He consumption) shows a maximum of 9.6 h for a neck length of 190 mm and He pot volume of 3.7 L (see Fig. 5C). Willing to minimize liquid He consumption, we decided to build the IVC with a neck of 270 mm and a He pot volume of 2.3 L. These dimensions guarantee a theoretical holding time of 8.1 h. In Fig. 5D, we report the corresponding temperature profile as a function of the distance from the He pot. The positions (90 mm and 180 mm) and consequent temperatures (90 K and 195 K) of two radiation shields are indicated on the graph by vertical and horizontal lines, respectively. The estimated liquid He consumption for this configuration is 0.284 L/h (i.e. 0.026 mol/s of gas flow).

Once determined the dimensions of the cryostat, it is interesting to use the calculated enthalpy variation that the gas experiences by flowing through the neck to evaluate the different heat loads affecting the behaviour of the cryostat. If from Eq. (7) we remove from the computation the probe contribution (i.e. the heat conduction happens only through the neck and He gas itself), the liquid He consumption for a cryostat with a neck of 270 mm and radiation shields mounted as described in the former paragraph, decreases to 0.092 L/h (see Supporting Information, Figure S2). This value corresponds to a He gas flow $\mu$ of 0.0009 mol/s. From $\Delta H=\mu \cdot {c}_{p}\cdot \left(294 K-4.2 K\right)$ we can calculate that the enthalpy variation (in Watt), thus the heat per second introduced by the probe, is 10.5 W. Instead, if we neglect completely the radiation, integrating Eq. (2) over a neck length of 270 mm and imposing the same boundary condition as before $\left|{T}_{end}-294\right|<0.5 K$, the He consumption decreases only to 0.240 L/h (0.0022 mol/s) (see Supporting Information, Figure S2). Therefore, in presence of radiation shields mounted as earlier described, the heat radiation contributes to the heat load with only 2.4 W.

We also investigate the possibility to decrease the He consumption by optimizing the position of the radiation shields for the chosen neck length. In this case, for a given set of value of the gas flow and temperature of shield 1 and shield 2, their distance from the He pot is varied in steps of 5 mm from 70 to 130 mm and from 170 to 230 mm, respectively. The condition of acceptance of the solution of Eq. (7) is on the resulting temperature value at the shield 1 position (${T}_{1}$), at the shield 2 position (${T}_{2}$), and at the top flange (${T}_{end}$): $\left|{T}_{1}-{T}_{shield1}\right|<0.5 K\wedge \left|{T}_{2}-{T}_{shield2}\right|<0.5 K\wedge \left|{T}_{end}-294\right|<0.5 K$.

**Figure 6:**
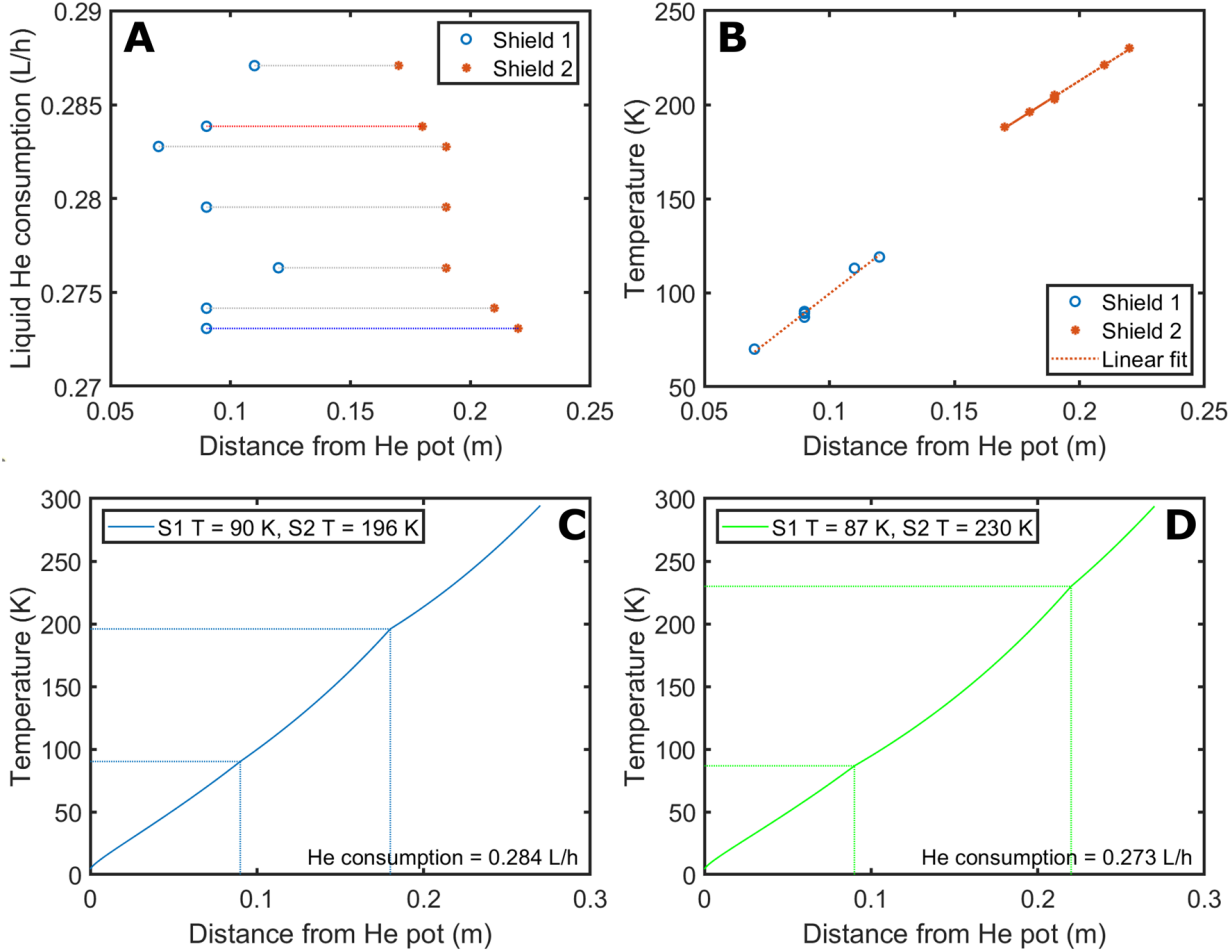
Liquid He consumption as a function of the shields position; the blue circles and the stars represent shield 1 and shield 2, respectively; shields positions leading to a solution of Eq. (4) for a given gas flow are connected by horizontal lines: the one in red corresponds to the shields places at 1/3 and 2/3 of the neck length, while the one is blue is the configuration providing the lowest consumption (**A**). Shield 1 and shield 2 temperatures as a function of their positions; in both cases data were fit using a linear relation (**B**). Temperature profile corresponding to the “non optimized” configuration (**C**, red line in **A**) and the best configuration (**D**, blue line in **A**).

We report the results of the radiation shields optimization in Fig. 6A. The blue circles indicate the position of the first radiation shield, while the red stars the position of the second. Horizontal dotted lines connect shields positions that provide an acceptable solution of Eq. (7) for a given gas flow. As a comparison, the configuration with the first shield at 1/3 of the cryostat neck length and the second at 2/3 is marked in red, while the one providing the lowest He consumption (i.e. 0.273 L/h) is marked in blue. The latter entails the first shield placed at 90 mm and the second at 220 mm. The temperature of the two shields increases linearly with the distance from the He pot (see Fig. 6B), but the two slopes are slightly different: 1.00 K/mm for shield 1 and 0.84 K/mm for shield 2. Most likely, this difference comes from the fact that, as explained in *Material and Methods*, the presence of the two radiation shields, and the corresponding heat input to the gas flow in correspondence of the anchoring position to the neck, imposes to solve Eq. (7) in 3 steps: from ${z= Z}_{0}$ to ${z= Z}_{1}$, from ${z= Z}_{1}$ to ${z= Z}_{2}$, and from ${z= Z}_{2}$ to ${Z=Z}_{max}$. Therefore, the solution is continuous because of the boundary conditions, but not its derivative. This can be easily seen in the temperature profile for the “non optimized” and “optimized” configuration of the screens, reported respectively in Fig. 6C,D, where in correspondence of the shields positions the curve presents two “edges”. Looking at Fig. 6A, it is interesting to notice that, for a given position of shield 1 (i.e. 90 mm) the He consumption decreases by moving shield 2 towards higher temperature. The latter increase the heat input by radiation between the two shields and reduces the one between the OVC and second shield. Similarly, for a given position of shield 2 (i.e. 190 mm) the He consumption decreases by moving shield 1 towards higher temperature. The latter increases the heat input by radiation between the first shield and the He pot and reduces the one between the second shield and the first shield. Since the Stefan-Boltzmann law increases with the 4th power of the temperature, in both cases, decreasing the contribution in the “higher temperature segment” is beneficial to reduce He consumption.

From a practical point of view, it is important to notice that, moving the radiation shields can improve the He consumption by only 5%, from 0.284 L/h (top horizontal line in Fig. 6A) to 0.273 L/h (bottom horizontal line in Fig. 6A). This is a consequence of the heavy heat load contribution from the NMR probe. Nevertheless, the presence of the shields remains a crucial design requirement. Indeed, no acceptable solution can be found for Eq. (7) if the radiation shields are omitted: a flow of 0.970 L/h provides solutions only from 190 mm onward (see Supporting Information, Figure S2).

### Cryogenic performance

Following the design indications resulting from the thermodynamic model, we built the cryostat with a neck length of 270 mm, a consequent reservoir capacity of 2.3 L (150 mm ID, 130 mm height), and with the centre of the heat exchanger of the two radiation shields placed at 90 mm and 220 mm above the top of the He pot.

**Figure 7:**
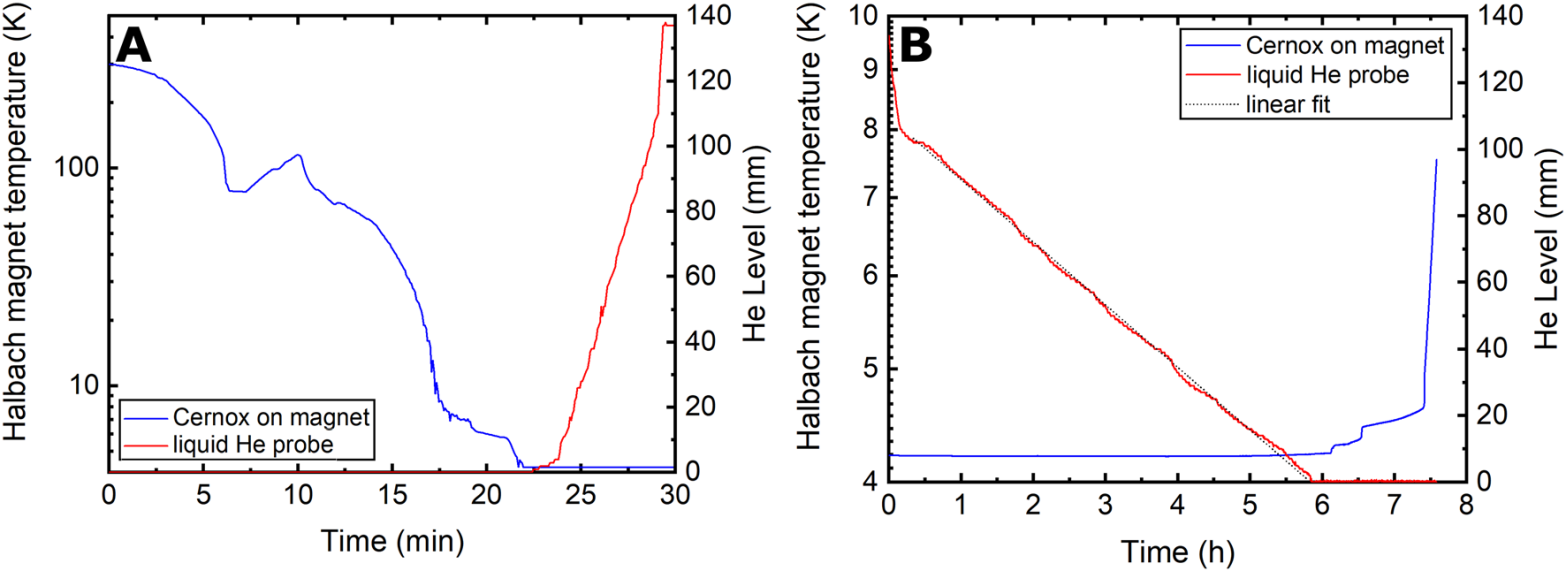
Cooldown and filling of the cryostat from room temperature to maximum liquid He capacity; the temperature of the magnet (blue line) and the He level (red line) were monitored (**A**). Holding time measurement after complete filling of the cryostat; the temperature of the magnet (blue line) and the He level (red line) were monitored; He consumption data were fit with a linear relation (**B**).

In Fig. 7A, we report the behaviour of the cryostat during cooldown from room temperature to liquid He temperature. The values read by the Cernox sensor as a function of time are indicated as a blue curve, while the He level reading as a red one. The precooling procedure using liquid N_2_ takes 5 min. Around this time point, we can observe a steep decrease of the temperature followed by a plateau that corresponds to N_2_ condensation around the temperature sensor. Right after, the temperature increases because we empty the pot from liquid N_2_ using He gas. As soon as we insert the liquid He syphon, the temperature starts to drop again reaching 4.2 K 17 min later, while keeping a constant overpressure of 5 mbar in the liquid He supply dewar. When the He starts to condense, we observe a second steep drop of the temperature and the He probe starts providing readings > 0 mm. It takes 5 more minutes to fill completely the He pot. The highest He level reading is 137 mm.

In Fig. 7B, we show how the cryostat performs during a typical “liquid He-holding experiment”. At the beginning, from 0 to 10 min, we observe a quick decrease (− 170 ± 5 mm/h) of the He level (red curve) until the reading reaches 110 ± 1 mm. Afterwards, the He consumption suddenly drops (− 18.7 ± 0.3 mm/h) and remains constant for 6 h, until the He level reaches the bottom of the copper plate attached to the storage magnet. During this time interval, the magnet stays at 4.2 K. Afterwards, we observe a slight temperature increase up to 4.5 K for 1.5 h more, because the Cernox temperature sensor gradually comes out from liquid He. Finally, the temperature increases abruptly when the cryostat is empty.

The ninefold higher consumption registered at the beginning is only apparent. Indeed, according to the cryostat and probe geometry, the top 27 mm of liquid He level reading are measured inside the neck (see Fig. 4). Here, with a useful cross section (neck cross section minus the space occupied by the probe) of 0.235 dm^2^, the He consumption calculates 0.380 ± 0.011 L/h. Once inside the He pot, with a useful cross section of 1.525 dm^2^, the liquid He consumption decreases to 0.285 ± 0.004 L/h, in good agreement with the thermodynamic model of the cryostat. Towards the end of the holding period, the temperature of shield 1 and shield 2 are approximately 90 K and 200 K (see Supporting Information, Figure S3).

Over repeated experiments, we estimated that the cryostat can keep the magnet at 4.2 K for 7.5 ± 0.2 h, and that approximately 5 L of liquid He are needed from the supply Dewar each time we want to cool down and fill it.

### Magnetic field performance

**Figure 8:**
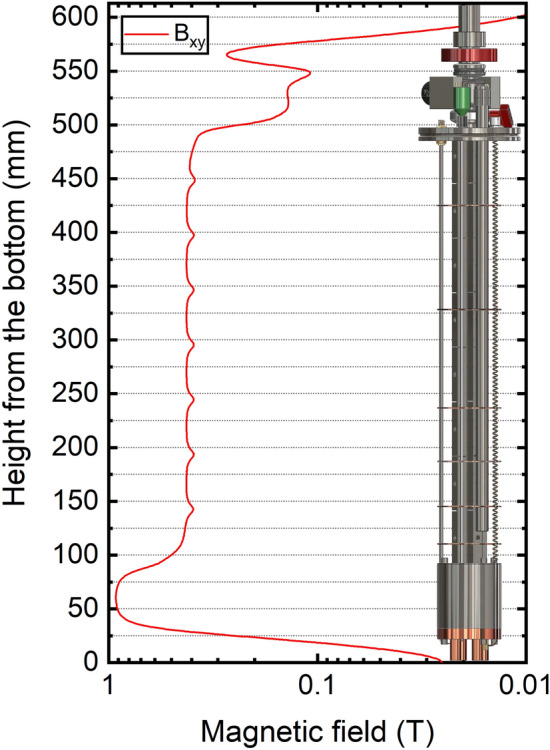
Simulation of the magnetic field profile along the vertical axis (*z*) of the magnetic probe. The direction of the magnetic field is in the xy-plane. A front view of cryogenic probe is superimposed to the plot to better correlate the magnetic field value to the cryogenic probe portion.

In Fig. 8, we show the result of the magnetic field simulation for the permanent magnets enforced NMR probe. The magnetic field values of the transversal field are calculated along the full length of the probe. A front view of the probe is superimposed to the graph to better correlate the magnetic field value to the probe region on the vertical axis. The permanent magnets arrangement provides, along the z axis, a magnetic field value of at least 0.100 T.

The lowest value is found between the two ISO-KF-16 flanges of the loading chamber and the gate valve. Below the ISO-K-63 flange, along the loading tube, the value oscillates between 0.420 T and 0.380 T, with minima in correspondence of the 1 mm PTFE spacer placed between the eight 50 mm long magnet stacks. The highest value of 0.964 m T appears in correspondence of the isocenter of the storage magnet at 60 mm from the bottom of the probe. The magnetic field simulations of the different Halbach arrays were checked using the Hall probe. Within 5% error, al results are in good agreement (e.g. measured field value of 0.936 T for the storage magnet vs simulated value of 0.964 T).

**Figure 9:**
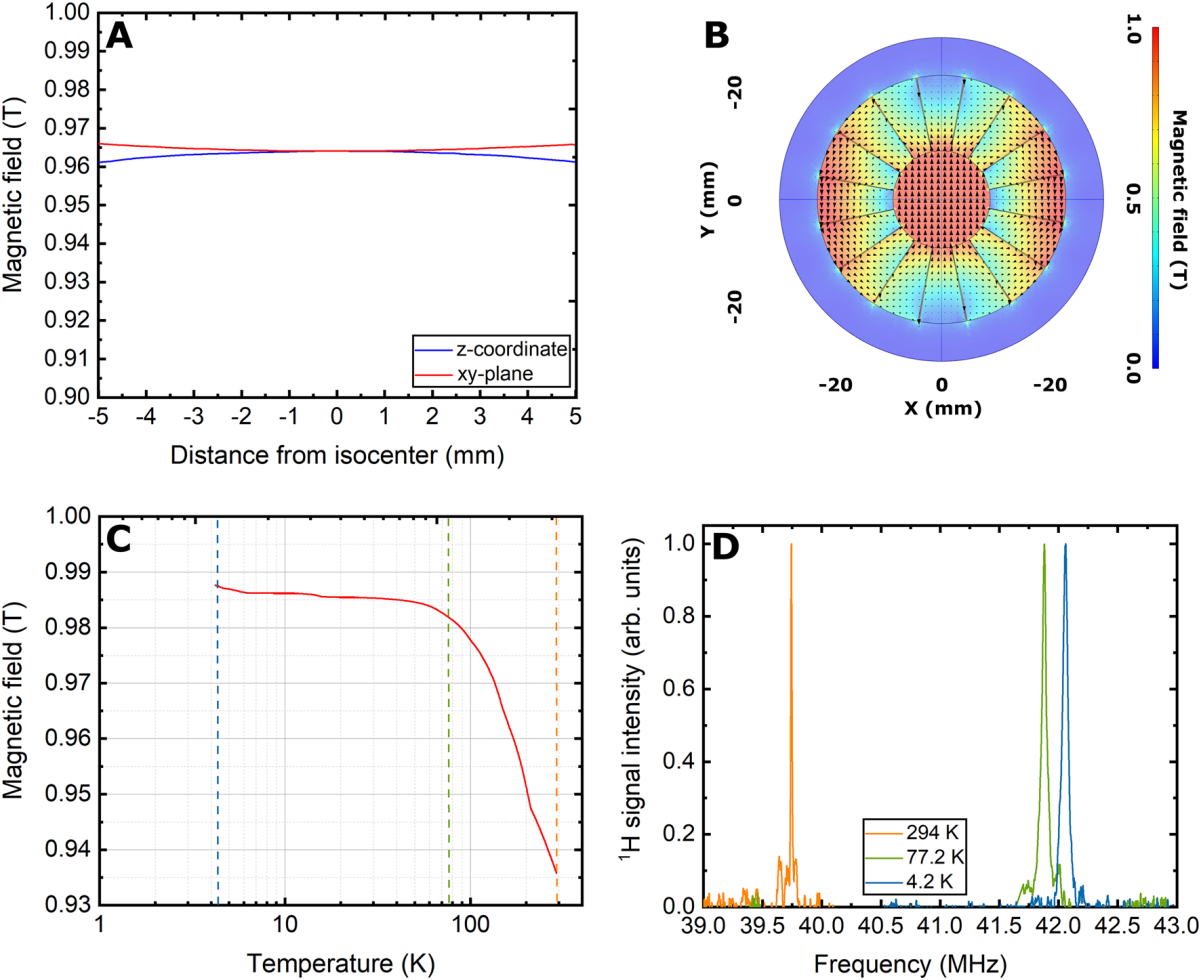
Magnetic field value simulation 10 mm across the storage magnet isocenter in the *z-*direction (blue curve) and *y-*direction (red curve); because of the magnet geometry, the solution evaluated on each diameter in the xy-plane is identical (**A**). 2D field map evaluated on transversal plane passing for the storage magnet isocenter (**B**). Storage magnet field drift as a function of temperature (red curve); measurements at 294 K, 7 K, and 4.2 K are indicated by the intersection with dashed lines in orange, green and blue colours, respectively (**C**). 1H NMR spectra collected at 294 K (orange), 77 K (green) and 4.2 K (blue) (**D**).

If magnetic field homogeneity is not very important to preserve the polarization when loading into the cryostat long relaxing HP samples^14^, it becomes a key requirement inside the storage magnet if we wish to perform NMR, especially in the absence of active shims. In Fig. 9A,B we report a more detailed picture of the magnetic field values calculated over 10 mm across the isocenter of the magnet in the *z-* and *y-*direction (the solution is symmetric in the xy-plane). With a central value of 0.9641 T and extreme values of 0.9620 T on the *z*-axis and 0.9659 mT on *y*-axis the homogeneity calculates 2178 ppm and 1867 ppm, respectively. In Fig. 9C, we report the measured permanent magnet behaviour as a function of temperature. From a value of 0.9361 T at room temperature (orange dashed line), the magnetic field increases steeply to 0.9819 T when cooling the magnet down to 77 K (green dashed line). Afterwards, the field increases slowly to 0.9871 T when reaching 4.2 K (blue dashed line). These three values were also checked with ^1^H NMR measuring on a water: glycerol solution doped with TEMPO (see *Materials and Methods*). The storage magnet at room, liquid N_2_ and liquid He provides resonances at 39.743 MHz, 41.881 MHz and 42.059 MHz, respectively (Fig. 9D). According to the amount of sample in the vial (125 μL), the NMR signal arises from a 5 mm x 5 mm x 5 mm volume. According to the full-width-at-half-maximum (FWHM) of 11.5 kHz, measured at room temperature, the corresponding magnetic field homogeneity is 289 ppm. At 77 K and 4.2 K the FWHW increases to 44.3 kHz and 51.9 kHz, respectively. These values are 4–5 times larger than the one measured at room temperature because of the magnet inhomogeneity. Therefore, the latter has minimal effect on the spectral appearance in the solid state, providing signals very similar to the ones that one can measure on the same sample from the DNP polarizer^32–36^. It is worth noticing that, over one year of use of the cryostat and repeated temperature cycles, the values of the NMR resonances at 4.2 K, 77 K and room temperature have never changed.

### Perspective to build a smaller transport device with limited holding time

The current transportation device weighs 15 kg and is 65 cm tall including the probe and loading chamber. A trolley is necessary to move it around in a comfortable way. Therefore, to simplify future applications that do not require liquid He holding time over 4—5 h, we investigate the possibility to build an even more compact and lighter weight transportation device.

We use the thermodynamic model to assess the feasibility of a single radiation shield cryostat with an IVC total length of 300 mm and a neck length of 200 mm. Consequently, the He pot capacity is 1.8 L (cylinder with ID of 150 mm and height 100 mm).

In this last case, the condition of acceptance of the solution is on the resulting temperature values at the shield position (${T}_{1}$) and at the top flange (${T}_{end}$): $\left|{T}_{1}-{T}_{shield1}\right|<0.5 K\wedge \left|{T}_{end}-294\right|<0.5 K$.

**Figure 10:**
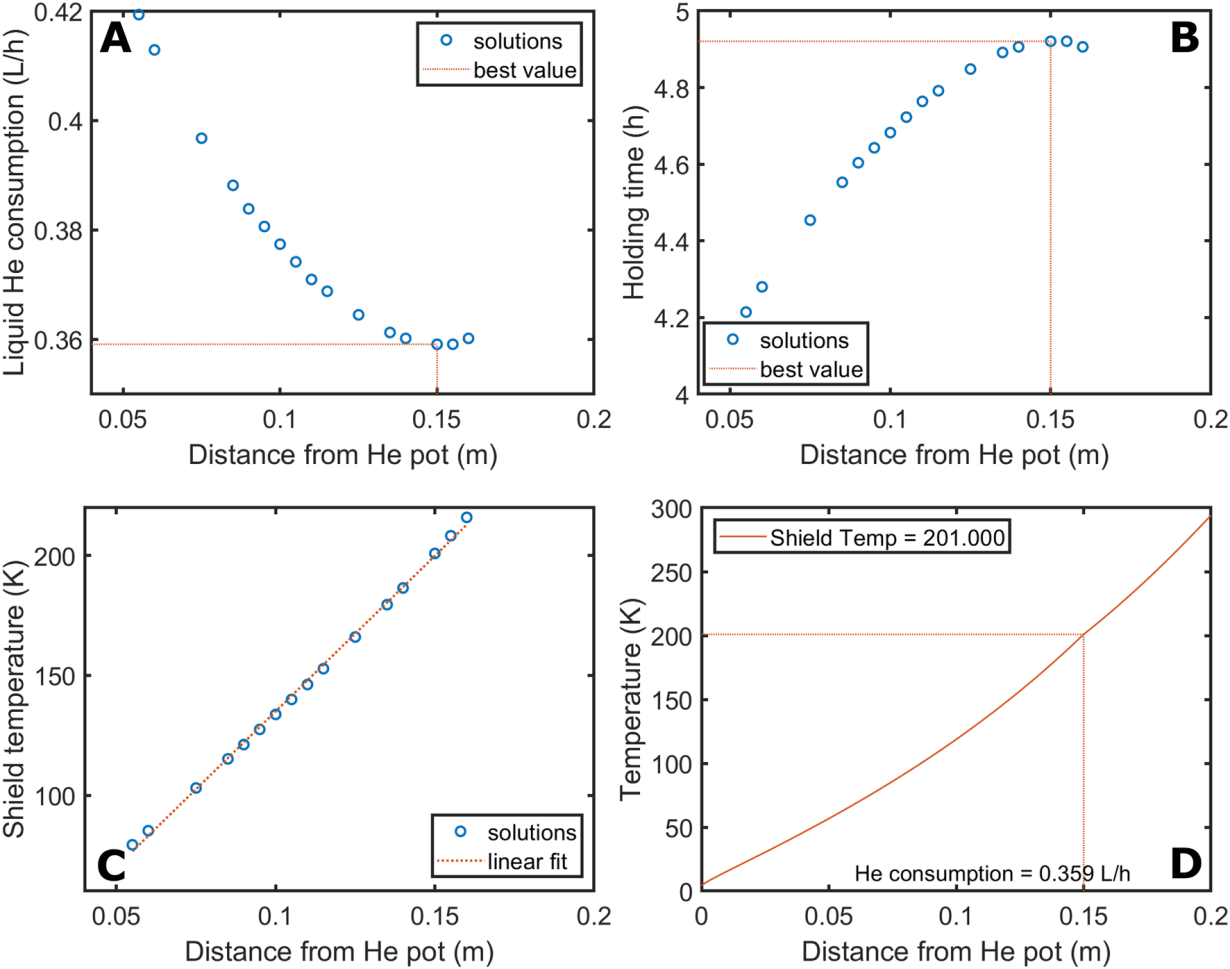
Liquid He consumption as a function of the radiation shield position over a neck length of 200 mm; the orange lines indicate the solution providing the lowest consumption (**A**). The corresponding holding time is reported in (**B**). Shield temperature as a function of its position; the dotted red line represents a linear fit (**C**). Temperature profile for the lowest consumption configuration; the horizontal and vertical lines indicate temperature and position of the radiation shield, respectively (**D**).

Figure 10A shows how the liquid He consumption changes as a function of the position of the radiation shield. Limiting the allowed He consumption to a maximum of 0.5 L/h, solution exist for a shield position between 55 and 160 mm from the He pot. The minimum appears at 150 mm with a consumption of 0.359 L/h. This position corresponds to a shield temperature of 201 K. This cryostat configuration would correspond to a holding time close to 5 h (see Fig. 10B). As in the former case, the temperature of the radiation shield increases linearly with the distance from the He pot with a slope of 1.3 K/mm (see Fig. 10C). Most likely, if compared to the former cryostat configuration, the steeper slope is a consequence of the reduced length of the neck for the same starting and ending temperatures. Figure 10D shows the temperature profile corresponding to the optimized position of the radiation shield.

### Link to previous works sharing similar hardware

We would like to conclude the discussion mentioning two works that, despite the different context, share some hardware similarities with what is herein described. In 2015, Dr James Kempf from Bruker Biospin led an initiative to produce transportable long relaxing samples hyperpolarized with the “brute force” method instead of DNP^21^. Although the intrinsically low ^13^C polarization (i.e. approximately 0.1%) and very long build-up time (i.e. tens of hours), due to the absence of radicals and microwave irradiation, the probe presented a He gas driven automatic sample extraction system pushing the HP solid through a magnetic tunnel connecting the polarizer and the storage/dissolution station. Similarly, Dr Benno Meier invented what is known as “bullet DNP” in 2019^37^. In that case the extraction of a HP solid sample still in presence of radicals, required a probe connected to 60 A powered solenoidal coil running until the receiving device, and a speed of 100 m/s to retain most of the spin order prior to dissolution. With no doubt, these two studies can be a source of inspiration and be combined with what we describe in this paper to get the best out of all setups and build a HP sample extraction/storage system as user-friendly as possible.

## Conclusions

In this paper, we report a detailed protocol to build an HP samples transportation device equipped with an NMR quality permanent magnets enforced cryogenic probe. Although we showed ^13^C NMR measurements of long relaxing HP samples obtained from this cryostat during the 2022 ISMRM conference^38^, we do not report them here because this subject will be covered in a future publication. We demonstrate the feasibility of a design that can hold liquid He for 7.5 h with a consumption of 0.285 L/h, and we provide the calculation for a smaller footprint version with a theoretical holding time close to 5 h.

We also perform a comprehensive investigation of the storage magnet field homogeneity and behaviour as a function of temperature. By means of proton NMR we show that the compact size 16 elements SmCo Halbach array can provide a magnetic field close to 1 T with a homogeneity of 289 ppm. The latter means that, once at low temperature, the NMR peak broadening coming from the nuclear dipolar coupling is predominant on the magnetic field inhomogeneity.

The work showed in this manuscript represents a first step towards transportable hyperpolarization and the proof that it is possible to build such a device. We are now investigating how to make the protocol more appealing, user-friendly, and less expensive on the long run. The latter will happen by perusing three main directions: to design a probe that can accommodate more than one sample, to make the sample transfer from the DNP polarizer to the storage device automatic and, most importantly, to upgrade the cryostat to a cryogen free version.

It is important to notice that, making hyperpolarization transportable directly addresses the limitation of needing a DNP polarizer on site to perform HP MRI. Other challenges such as the quest for performant X-nuclei coils, specialized sequences and electronics (e.g. stronger gradients), that are currently limiting wide adoption of HP MRI in the clinics, still hold. Nevertheless, making hyperpolarization more available in a PET-like model will increase the number of sites in need of X-nuclei specialized tools, initiating, most likely, a common and broader effort in that direction.

Should transportable hyperpolarization become a reality, we herein provide the MR community with a strategy and many key elements to make it possible and fill the technical gap with transported PET tracers.

## Supplementary Information

Supplementary Information.

## Data Availability

The author declares that all data supporting the findings of this study are available within the paper and its supplementary information files. Raw data are available from the corresponding author (andrea.capozzi@epfl.ch) on reasonable request.
