# Supplementary material for: Design and performance of a small bath cryostat with NMR capability for transport of hyperpolarized samples
Source: Sci Rep. 2022 Nov 10;12:19260. doi: 10.1038/s41598-022-23890-7 (PMC9649762; doi:10.1038/s41598-022-23890-7)
Supplement: Supplementary file 1 — Supplementary Information. [file 41598_2022_23890_MOESM1_ESM.docx]

**Supporting Information**

**Design and performance of a small bath cryostat with NMR capability for transport of hyperpolarized samples.**

Andrea Capozzi ^1,2^

*^1^LIFMET, Institute of Physics, EPFL, Station 6 (Bâtiment CH), 1015 Lausanne (Switzerland).*

*^2^HYPERMAG, Department of Health Technology, Technical University of Denmark, Building 349, 2800 Kgs Lyngby (Denmark).*

**1. View factor**

A geometrical view factor *F_12_* is defined as the fraction of the total radiation leaving the first body that is intercepted and absorbed by the second. View factors depend on the relative orientation of the two surfaces involved ^1^. View factors are tedious to calculate, but most common values can be found in the literature. As far as we are concerned in this paper, we used the expression that more resembles to a radiation shield facing the other radiation shield or the OVC/IVC: two finite length cylinders with the interior surface of the outer cylinder emitting towards the outer surface of the inner cylinder. The expression that follows was found here (<http://www.cfdyna.com/Notes/ViewFactors.pdf>) and used in Equation (2) in the main text:

$F_{n+1\to n}=\frac{1}{\pi R_{n+1}}\left[ \frac{1}{2}\left( R_{n+1}-R_{n}-1 \right){cos}^{-1}\left( \frac{R_{n}}{R_{n+1}} \right)+\pi R_{n}-\frac{\pi}{2}AB-2R_{n}{tan}^{-1}\left( {R_{n+1}}^{2}-{R_{n}}^{2} \right)^{\frac{1}{2}}+\left( \left( 1+A^{2} \right)+\left( 1+B^{2} \right) \right)^{1/2}{tan}^{-1}\left( \frac{\left( 1+A^{2} \right)B}{\left( 1+B^{2} \right)A} \right)^{1/2} \right]$

with $R_{n}={r_{n}}/h, R_{n+1}={r_{n+1}}/h, A=R_{n+1}+R_{n}, B=R_{n+1}-R_{n}$; where $r_{n+1}$ is the radius of the external cylinder, $r_{n}$ is the radius of the internal cylinder and $h$ is the height. Being the formula for cylinders of the same height (not exactly the case for a cryostat), in the model we assumed as $h$ the average between the heights of the two cylindrical surfaces involved.

**2. Temperature dependent thermal conductivity**

As mentioned in the main text, the temperature dependent conductivity for stainless steel and He gas, used to solve Equation 3 in the main text, were obtained by polynomial fits of data tables ^2,3^. In Figure S1 you can find the result. It is important to notice that the stainless steel conductivity is 2 orders of magnitude higher than the He gas one.


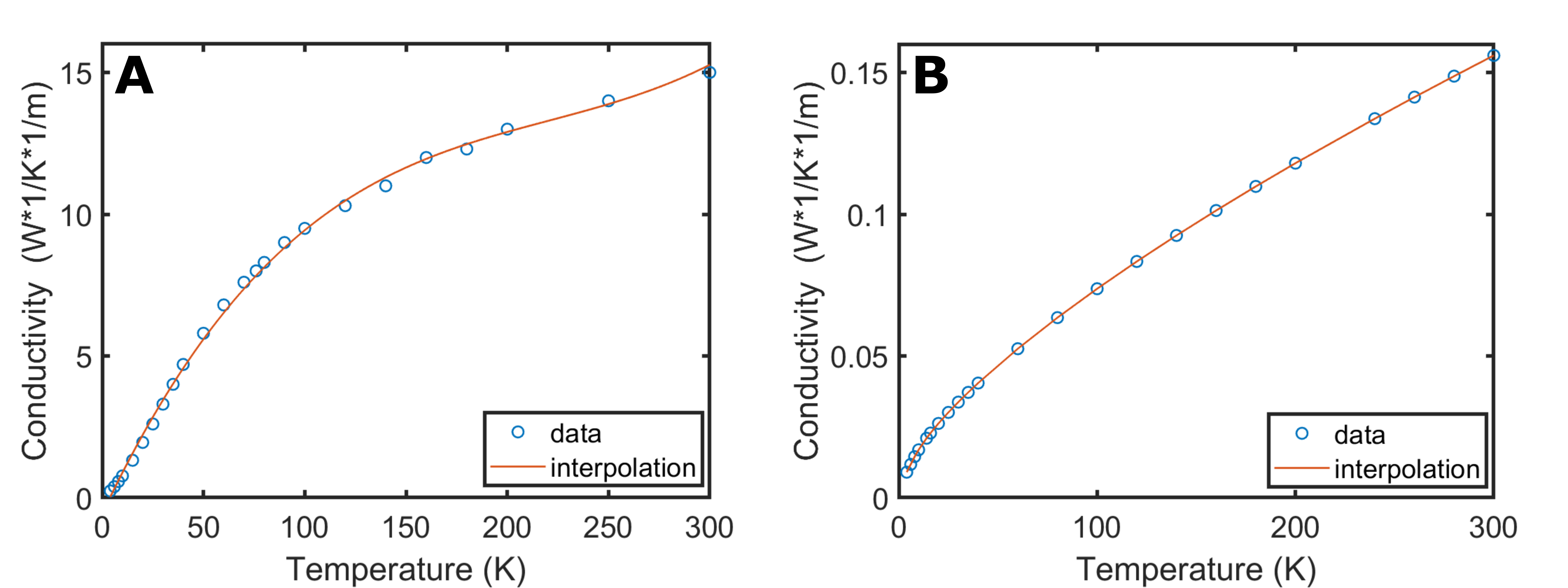


**Figure S1.** Third degree polynomial interpolation of the thermal conductivity data for stainless steel (**A**) and He gas (**B**).

**3. Evaluation of different heat loads on over liquid He consumption**


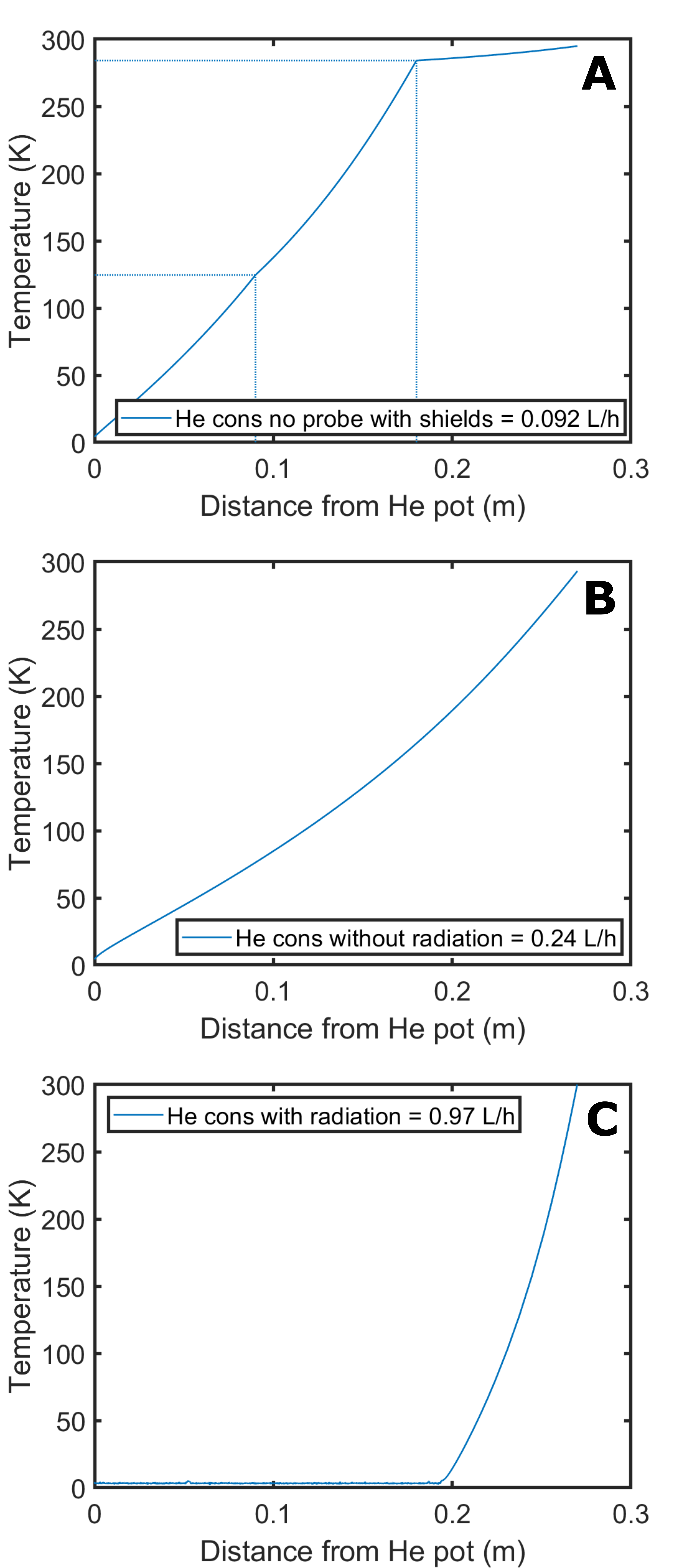


**Figure S2.** We report here plots of additional results described in paragraph 3.1 concerning the evaluation of the different heat loads in the cryostat. We show the calculated liquid He consumption for the case of cryostat without the NMR probe (**A**); without the heat load from the radiation radiation (**B**); without the radiation shields (**C**).

**4. Radiation shields temperature measurement**

**
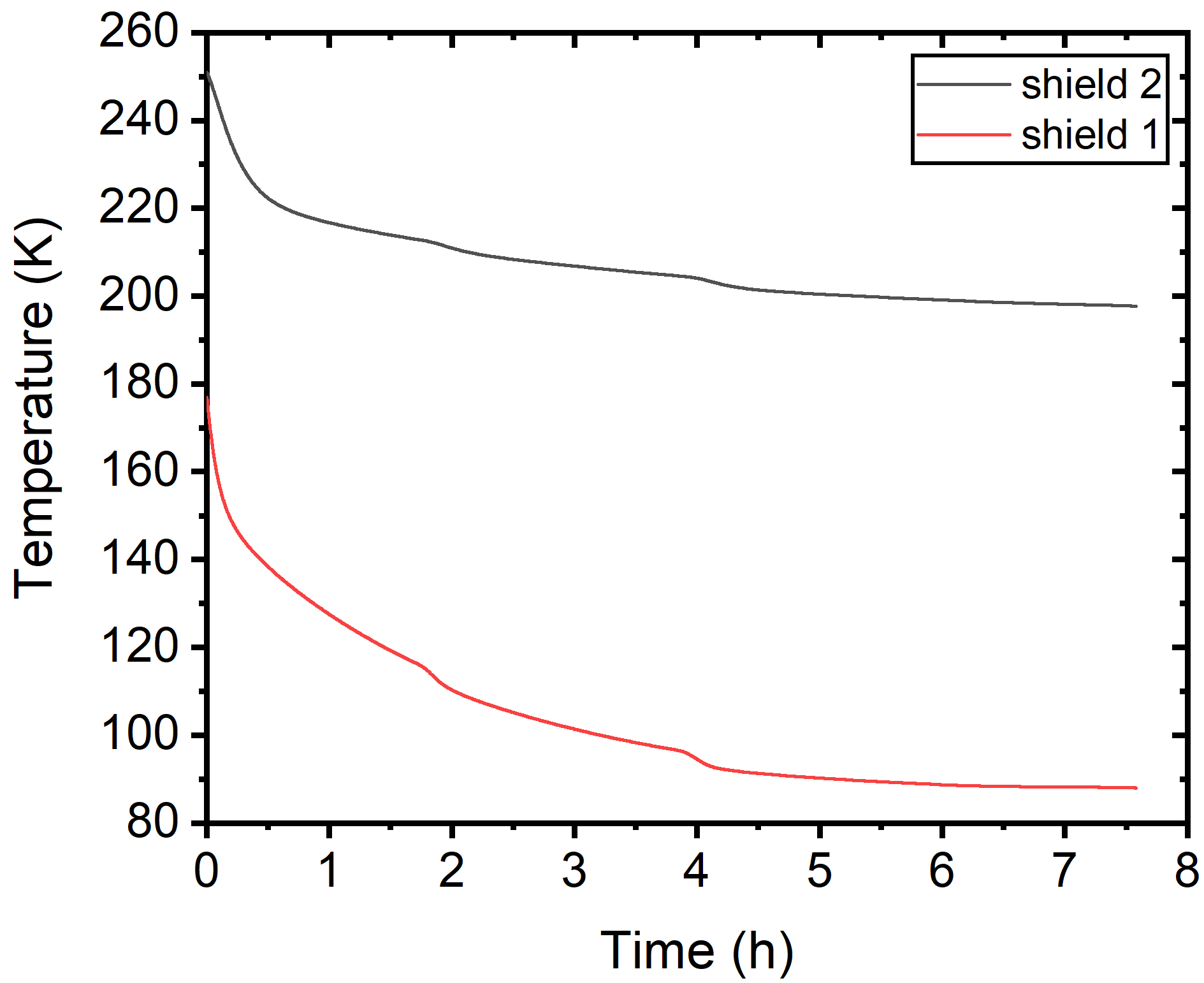
**

**Figure S3.** Measurement of the temperature evolution over time of shield 1 (red) and shield 2 (black) during a holding experiment.

**5. MATLAB code**

**5.1 No shields case**

%This is a thermodynamic model to estimate the liquid He consumption in a

%small bath cryostat in absence of radiation shields

%the bath is at 4.2K

%the aim is to find a temperature profile as a function of the distance from the He bath for a given gas flow

clear all

close all

%First of all, we consider only the contribution to the heat transfer of

%the cryostat metal parts: neck and probe (conduction)

%we assume that all is stainless steel

cross_section_neck = pi*((0.0635/2)^2-(0.0625/2)^2);

cross_section_tube = pi*((0.013/2)^2-(0.012/2)^2);

cross_section_square = 0.01^2-0.008^2;

cross_section_syphon = pi*((0.0115/2)^2-(0.0105/2)^2);

cross_section_coax = pi*((0.0032/2)^2-(0.003/2)^2);

cross_section_He_probe = pi*0.001^2;

cross_section = cross_section_He_probe + cross_section_coax + cross_section_neck + cross_section_tube + 4*cross_section_square +...

cross_section_syphon; %m^2

%let's determine the thermal conductivity function of SS304L from experimental

%data. Conductivity is in W*m^-1*K^-1

temp_list_304L = [4, 6, 8, 10, 15, 20, 25, 30, 35, 40, 50, 60, 70, 76, 80,...

90, 100, 120, 140, 160, 180, 200, 250, 300];

cond_list_304L = [0.24, 0.39, 0.57, 0.77, 1.32, 1.95, 2.60, 3.30,...

4.00, 4.70, 5.80, 6.80, 7.60, 8.00, 8.30, 9.00, 9.50, 10.30, 11.00, 12.00, 12.30, 13.00, 14.00, 15.00];

temperature = 4:0.1:300;

cond_304L = interp1(temp_list_304L, cond_list_304L, temperature);

%let's define flow and molar enthalpy: for each z the enthalpy flow is

%flow*H(T) = flow*cp = flow*5/2*R = flow*20.7 J*mol^-1*K^-1

%consideration on the flow: in 1 mole we have 22.4 L; 1 lit of liquid

%helium corresponds to 757 lit of gas; we measure a boil-off rate of 0.3

%L/h ==> 0.0631 lit/s ==> 0.0028 mol/s

cp = 20.7; %J*mol^-1*K^-1

%the equation to solve is flow*cp*T = cross_section*K(T)*dT/dz. For Ode45

%you have to explicit the dT/dz term

%let's get the polynomial expression that approx K_304L(T), we use order 3

K_T_304L = polyfit(temperature, cond_304L, 3);

%Let's solve the differential equation: initial value is T = 4.2 K and the

%we find solution for all the length of the neck (0 ---> 0.27 m)

cryostat_neck = 0.27;

T0 = 4.2;

%lets now introduce the radiation from the shields : Pn(W) =

%emiss_n*Stef_const*Tn^4.

Stef_const = 5.67e-8; %W*m^-2*K^-4

%I write down the emissivity of Al at the

%interesting temperatures

emiss_Al_4K = 0.04;

emiss_SS_RT = 0.2;

%I write down all important dimensions of shields, He pot, and OVC. All is

%in meters

He_pot_r = 0.154/2;

He_pot_h = 0.138;

OVC_r = 0.197/2;

OVC_h = 0.450;

%I calculate all cylindrical surfaces

He_pot_surf = shield_surface(He_pot_r, He_pot_h);

OVC_surf = shield_surface(OVC_r, OVC_h);

%I calculate all view factors. The view factor found in the literature

%assumes equal height of the cylinders. We have different values. We put

%the mean to compensate

vf_pot_OVC = view_factor(He_pot_r, OVC_r, (He_pot_h+OVC_h)/2);

%for a pair of screens at temperature T1 and T2 with T1<T1 the power (W) dumped at the

%junction between 1 and the central tube is q_1_2 = Stef_const*(surf1*(T2^4-T1^4)*F12)/(1/eps1+surf1/surf2*(1/eps1-1))

%the function radiation(T1, eps1, S1, T2, eps2, S2, F12) calculates this. I

%do it for the 3 cases of He_pot to shield 1; shield_1 to shield_2;

%shield_2 to OVC

q_He_pot_OVC = radiation(4.2, emiss_Al_4K, He_pot_surf, 294, emiss_SS_RT, OVC_surf, vf_pot_OVC);

%now we add the contribution of the heat conduction through the column of gas itself

% from the CRC Handbook we find the thermal conductivity of He gas as a

% function of temperature W*m^-1*K^-1

temp_list_He = [4, 6, 8, 10, 14, 16, 20, 25, 30, 35, 40, 60, 80, 100, 120, 140, 160, 180,...

200, 240, 260, 280, 300];

cond_list_He = 0.001*[9.004, 11.74, 14.49, 16.89, 20.98, 22.81, 26.20, 30.09, 33.72, 37.15,...

40.44, 52.55, 63.52, 73.71, 83.33, 92.5, 101.3, 109.8, 118.0, 133.7, 141.3, 148.7, 156.0];

%we interpolate again

cond_He = interp1(temp_list_He, cond_list_He, temperature);

%let's get the polynomial law that approx K_He(T), we use order 3

K_T_He = polyfit(temperature, cond_He, 3);

%He column cross section is the ID of the neck

cross_section_He = pi*(0.0625/2)^2;

%first of all I find the flow that allows me to reach 100 K after 9 cm

flow_guess1 = 0:0.000001:0.01; %mol/s

z_span1 = [0 0.27];

z_span2 = [0 0.27];

%Here we consider the case of heat input from conduction of the probe,

%cryostat neck and He gas column

for m = 1:length(flow_guess1)

F1 = @(z,T) (flow_guess1(m)*cp*T)/((1.8*cross_section*(K_T_304L(1)*T^3+K_T_304L(2)*T^2+K_T_304L(3)*T+K_T_304L(4)))+...

(cross_section_He*((K_T_He(1)*T^3+K_T_He(2)*T^2+K_T_He(3)*T+K_T_He(4)))));

[z1,T_1] = ode45(F1, z_span1, T0);

if abs(T_1(end) - 294) < 1

flow_guess1(m);

break

end

end

flow1 = flow_guess1(m);

figure

plot(z1,T_1,'linewidth', 1);

box on

set(gca,'FontSize',16);

set(gca,'linewidth',2)

xlim([0 0.3]);

ylim([0 300]);

xlabel('Distance from He pot (m)');

ylabel('Temperature (K)');

legend(append('He cons without radiation = ', num2str(flow1*107.5, '%.2f'), ' L/h'), 'Location', 'northwest');

%Here we consider the case of heat input from conduction of the probe,

%cryostat neck, He gas column and radiation from OVC to IVC

flow_guess2 = 0.009:0.000001:0.0095;

for j = 1:length(flow_guess2)

F2 = @(z,T) (flow_guess2(j)*cp*T - q_He_pot_OVC)/((1.8*cross_section*(K_T_304L(1)*T^3+K_T_304L(2)*T^2+K_T_304L(3)*T+K_T_304L(4)))+...

(cross_section_He*((K_T_He(1)*T^3+K_T_He(2)*T^2+K_T_He(3)*T+K_T_He(4)))));

[z2,T_2] = ode45(F2, z_span2, T0);

if abs(T_2(end) - 294) < 10

break

end

end

flow2 = flow_guess2(j);

figure

plot(z2,T_2,'linewidth', 1);

box on

set(gca,'FontSize',16);

set(gca,'linewidth',2)

xlim([0 0.3]);

ylim([0 300]);

xlabel('Distance from He pot (m)');

ylabel('Temperature (K)');

legend(append('He cons with radiation = ', num2str(flow2*107.54, '%.2f'), ' L/h'), 'Location', 'northwest');

%Here we consider the case of heat input from conduction of cryostat neck and He gas column

for i = 1:length(flow_guess1)

F1 = @(z,T) (flow_guess1(i)*cp*T)/((cross_section_neck*(K_T_304L(1)*T^3+K_T_304L(2)*T^2+K_T_304L(3)*T+K_T_304L(4)))+...

(cross_section_He*((K_T_He(1)*T^3+K_T_He(2)*T^2+K_T_He(3)*T+K_T_He(4)))));

[z3,T_3] = ode45(F1, z_span1, T0);

if abs(T_3(end) - 294) < 1

flow_guess1(i);

break

end

end

flow3 = flow_guess1(i);

figure

plot(z3,T_3,'linewidth', 1);

box on

set(gca,'FontSize',16);

set(gca,'linewidth',2)

xlim([0 0.3]);

ylim([0 300]);

xlabel('Distance from He pot (m)');

ylabel('Temperature (K)');

legend(append('He cons no rad no probe = ', num2str(flow3*107.5, '%.2f'), ' L/h'), 'Location', 'northwest');

%Here we do some tests on the accuracy of the interpolation of the

%conductance data

cond_304L_short = interp1(temp_list_304L, cond_list_304L, T_1);

cond_He_short = interp1(temp_list_He, cond_list_He, T_1);

cond_304L_test = [];

cond_He_test = [];

for a = 1:length(T_1)

cond_304L_test = [cond_304L_test, (K_T_304L(1)*T_1(a)^3+K_T_304L(2)*T_1(a)^2+K_T_304L(3)*T_1(a)+K_T_304L(4))];

cond_He_test = [cond_He_test, (K_T_He(1)*T_1(a)^3+K_T_He(2)*T_1(a)^2+K_T_He(3)*T_1(a)+K_T_He(4))];

end

figure

plot(temp_list_He, cond_list_He, 'o', 'linewidth', 1);

hold on

plot(temperature, cond_He, 'linewidth', 1);

box on

set(gca,'FontSize',16);

set(gca,'linewidth',2)

xlim([0 300]);

ylim([0 0.16]);

xlabel('Temperature (K)');

ylabel('Conductivity (W*1/K*1/m)');

legend('data', 'interpolation');

cond_fit_304L = polyval(K_T_304L, temperature);

figure

plot(temp_list_304L, cond_list_304L, 'o', 'linewidth', 1);

hold on

plot(temperature, cond_fit_304L, 'linewidth', 1);

box on

set(gca,'FontSize',16);

set(gca,'linewidth',2)

xlim([0 300]);

ylim([0 16]);

xlabel('Temperature (K)');

ylabel('Conductivity (W*1/K*1/m)');

legend('data', 'interpolation');

**5.2 Determination of optimal neck length, radiation shields always at 1/3 and 2/3 of the neck length**

%This is a thermodynamic model to evaluate the consumption of a small bath

%cryostat as a function of the length of the neck

%the bath is at 4.2K; the 2 radiation shields are always at 1/3 and 2/3 of the neck length

%the aim is to find a temperature profile as a function of the distance from the He bath for a given gas flow

%%

clear all

close all

%First of all, we consider only the contribution to the heat transfer of

%the cryostat metal parts: neck and probe (conduction)

%we assume that all is stainless steel

cross_section_neck = pi*((0.0635/2)^2-(0.0625/2)^2);

cross_section_tube = pi*((0.013/2)^2-(0.012/2)^2);

cross_section_square = 0.01^2-0.008^2;

cross_section_syphon = pi*((0.0115/2)^2-(0.0105/2)^2);

cross_section_coax = pi*((0.0032/2)^2-(0.003/2)^2);

cross_section_He_probe = pi*0.001^2;

cross_section = cross_section_He_probe + cross_section_coax + cross_section_neck + cross_section_tube + 4*cross_section_square +...

cross_section_syphon; %m^2

%let's determine the thermal conductivity function of SS304L from experimental

%data. Conductivity is in W*m^-1*K^-1

temp_list_304L = [4, 6, 8, 10, 15, 20, 25, 30, 35, 40, 50, 60, 70, 76, 80,...

90, 100, 120, 140, 160, 180, 200, 250, 300];

cond_list_304L = [0.24, 0.39, 0.57, 0.77, 1.32, 1.95, 2.60, 3.30,...

4.00, 4.70, 5.80, 6.80, 7.60, 8.00, 8.30, 9.00, 9.50, 10.30, 11.00, 12.00, 12.30, 13.00, 14.00, 15.00];

temperature = 4:0.1:300;

cond_304L = interp1(temp_list_304L, cond_list_304L, temperature);

%let's define flow and molar enthalpy: for each z the enthalpy flow is

%flow*H(T) = flow*cp = flow*5/2*R = flow*20.7 J*mol^-1*K^-1

%consideration on the flow: in 1 mole we have 22.4 L; 1 lit of liquid

%helium corresponds to 757 lit of gas; we measure a boil-off rate of 0.3

%L/h ==> 0.0631 lit/s ==> 0.0028 mol/s

cp = 20.7; %J*mol^-1*K^-1

%the equation to solve is flow*cp*T = cross_section*K(T)*dT/dz. For Ode45

%you have to explicit the dT/dz term

%let's get the polynomial expression that approx K_304L(T), we use order 3

K_T_304L = polyfit(temperature, cond_304L, 3);

%Let's solve the differential equation: initial value is T = 4.2 K and the

%we find solution for all the length of the neck (0 ---> 0.27 m)

T0 = 4.2;

%lets now introduce the radiation from the shields : Pn(W) =

%emiss_n*Stef_const*Tn^4.

Stef_const = 5.67e-8; %W*m^-2*K^-4

%I write down the emissivity of Al at the

%interesting temperatures

emiss_Al_4K = 0.04;

emiss_Al_100K = 0.08;

emiss_Al_200K = 0.1;

emiss_SS_RT = 0.2;

%I write down all important dimensions of shields, He pot, and OVC. All is

%in meters

He_pot_r = 0.154/2;

He_pot_h = 0.138;

shield_1_r = 0.175/2;

shield_1_h = 0.235;

shield_2_r = 0.185/2;

shield_2_h = 0.340;

OVC_r = 0.197/2;

OVC_h = 0.450;

%I calculate all cylindrical surfaces

He_pot_surf = shield_surface(He_pot_r, He_pot_h);

shield_1_surf = shield_surface(shield_1_r, shield_1_h);

shield_2_surf = shield_surface(shield_2_r, shield_2_h);

OVC_surf = shield_surface(OVC_r, OVC_h);

%I calculate all view factors. The view factor found in the literature

%assumes equal height of the cylinders. We have different values. We put

%the mean to compensate

vf_pot_shiled_1 = view_factor(He_pot_r, shield_1_r, (He_pot_h+shield_1_h)/2);

vf_shiled_1_shiled_2 = view_factor(shield_1_r, shield_2_r, (shield_1_h+shield_2_h)/2);

vf_shiled_2_OVC = view_factor(shield_2_r, OVC_r, (shield_2_h+OVC_h)/2);

%for a pair of screens at temperature T1 and T2 with T1<T1 the power (W) dumped at the

%junction between 1 and the central tube is q_1_2 = Stef_const*(surf1*(T2^4-T1^4)*F12)/(1/eps1+surf1/surf2*(1/eps1-1))

%the function radiation(T1, eps1, S1, T2, eps2, S2, F12) calculates this. I

%do it for the 3 cases of He_pot to shield 1; shield_1 to shield_2;

%shield_2 to OVC

q_He_pot_shield_1 = radiation_2(emiss_Al_4K, He_pot_surf, emiss_Al_100K, shield_1_surf, vf_pot_shiled_1);

q_shield_1_shield_2 = radiation_2(emiss_Al_100K, shield_1_surf, emiss_Al_200K, shield_2_surf, vf_shiled_1_shiled_2);

q_shield_2_OVC = radiation_2(emiss_Al_200K, shield_2_surf, emiss_SS_RT, OVC_surf, vf_shiled_2_OVC);

%now we add the contribution of the heat conduction through the column of gas itself

% from the CRC Handbook we find the thermal conductivity of He gas as a

% fucntion of temperature W*m^-1*K^-1

temp_list_He = [4, 6, 8, 10, 14, 16, 20, 25, 30, 35, 40, 60, 80, 100, 120, 140, 160, 180,...

200, 240, 260, 280, 300];

cond_list_He = 0.001*[9.004, 11.74, 14.49, 16.89, 20.98, 22.81, 26.20, 30.09, 33.72, 37.15,...

40.44, 52.55, 63.52, 73.71, 83.33, 92.5, 101.3, 109.8, 118.0, 133.7, 141.3, 148.7, 156.0];

%we interpolate again

cond_He = interp1(temp_list_He, cond_list_He, temperature);

%let's get the polynomial law that approx K_He(T), we use order 3

K_T_He = polyfit(temperature, cond_He, 3);

%He column cross section is the ID of the neck

cross_section_He = pi*(0.0625/2)^2;

%first of all I find the flow that allows me to reach 100 K after 9 cm

flow_guess = 0.002:0.00001:0.008; %mol/s

cryostat_neck_guess = 0.105:0.005:0.40; %m

colors = ['#0072BD'; '#D95319' ; '#EDB120'; '#7E2F8E'; '#77AC30'; '#4DBEEE'; '#A2142F'; '#FF0000'; '#00FF00'; '#0000FF'; '#00FFFF'; '#D95319'; '#00FFFF';...

'#0072BD'; '#D95319' ; '#EDB120'; '#7E2F8E'; '#77AC30'; '#4DBEEE'; '#A2142F'; '#FF0000'; '#00FF00'; '#0000FF'; '#00FFFF'; '#D95319'; '#00FFFF'; '#0072BD'; '#D95319' ; '#EDB120'; '#7E2F8E'; '#77AC30'; '#4DBEEE'; '#A2142F'; '#FF0000'; '#00FF00'; '#0000FF'; '#00FFFF'; '#D95319'; '#00FFFF';...

'#0072BD'; '#D95319' ; '#EDB120'; '#7E2F8E'; '#77AC30'; '#4DBEEE'; '#A2142F'; '#FF0000'; '#00FF00'; '#0000FF'; '#00FFFF'; '#D95319'; '#00FFFF'; '#0072BD'; '#D95319' ; '#EDB120'; '#7E2F8E'; '#77AC30'; '#4DBEEE'; '#A2142F'; '#FF0000'; '#00FF00'; '#0000FF'; '#00FFFF'; '#D95319'; '#00FFFF';...

'#0072BD'; '#D95319' ; '#EDB120'; '#7E2F8E'; '#77AC30'; '#4DBEEE'; '#A2142F'; '#FF0000'; '#00FF00'; '#0000FF'; '#00FFFF'; '#D95319'; '#00FFFF'; '#0072BD'; '#D95319' ; '#EDB120'; '#7E2F8E'; '#77AC30'; '#4DBEEE'; '#A2142F'; '#FF0000'; '#00FF00'; '#0000FF'; '#00FFFF'; '#D95319'; '#00FFFF';...

'#0072BD'; '#D95319' ; '#EDB120'; '#7E2F8E'; '#77AC30'; '#4DBEEE'; '#A2142F'; '#FF0000'; '#00FF00'; '#0000FF'; '#00FFFF'; '#D95319'; '#00FFFF';'#0072BD'; '#D95319' ; '#EDB120'; '#7E2F8E'; '#77AC30'; '#4DBEEE'; '#A2142F'; '#FF0000'; '#00FF00'; '#0000FF'; '#00FFFF'; '#D95319'; '#00FFFF';...

'#0072BD'; '#D95319' ; '#EDB120'; '#7E2F8E'; '#77AC30'; '#4DBEEE'; '#A2142F'; '#FF0000'; '#00FF00'; '#0000FF'; '#00FFFF'; '#D95319'; '#00FFFF'; '#0072BD'; '#D95319' ; '#EDB120'; '#7E2F8E'; '#77AC30'; '#4DBEEE'; '#A2142F'; '#FF0000'; '#00FF00'; '#0000FF'; '#00FFFF'; '#D95319'; '#00FFFF';...

'#0072BD'; '#D95319' ; '#EDB120'; '#7E2F8E'; '#77AC30'; '#4DBEEE'; '#A2142F'; '#FF0000'; '#00FF00'; '#0000FF'; '#00FFFF'; '#D95319'; '#00FFFF';'#0072BD'; '#D95319' ; '#EDB120'; '#7E2F8E'; '#77AC30'; '#4DBEEE'; '#A2142F'; '#FF0000'; '#00FF00'; '#0000FF'; '#00FFFF'; '#D95319'; '#00FFFF';...

'#0072BD'; '#D95319' ; '#EDB120'; '#7E2F8E'; '#77AC30'; '#4DBEEE'; '#A2142F'; '#FF0000'; '#00FF00'; '#0000FF'; '#00FFFF'; '#D95319'; '#00FFFF'; '#0072BD'; '#D95319' ; '#EDB120'; '#7E2F8E'; '#77AC30'; '#4DBEEE'; '#A2142F'; '#FF0000'; '#00FF00'; '#0000FF'; '#00FFFF'; '#D95319'; '#00FFFF';...

'#0072BD'; '#D95319' ; '#EDB120'; '#7E2F8E'; '#77AC30'; '#4DBEEE'; '#A2142F'; '#FF0000'; '#00FF00'; '#0000FF'; '#00FFFF'; '#D95319'; '#00FFFF';'#0072BD'; '#D95319' ; '#EDB120'; '#7E2F8E'; '#77AC30'; '#4DBEEE'; '#A2142F'; '#FF0000'; '#00FF00'; '#0000FF'; '#00FFFF'; '#D95319'; '#00FFFF';...

'#0072BD'; '#D95319' ; '#EDB120'; '#7E2F8E'; '#77AC30'; '#4DBEEE'; '#A2142F'; '#FF0000'; '#00FF00'; '#0000FF'; '#00FFFF'; '#D95319'; '#00FFFF'; '#0072BD'; '#D95319' ; '#EDB120'; '#7E2F8E'; '#77AC30'; '#4DBEEE'; '#A2142F'; '#FF0000'; '#00FF00'; '#0000FF'; '#00FFFF'; '#D95319'; '#00FFFF';...

'#0072BD'; '#D95319' ; '#EDB120'; '#7E2F8E'; '#77AC30'; '#4DBEEE'; '#A2142F'; '#FF0000'; '#00FF00'; '#0000FF'; '#00FFFF'; '#D95319'; '#00FFFF'; '#0072BD'; '#D95319' ; '#EDB120'; '#7E2F8E'; '#77AC30'; '#4DBEEE'; '#A2142F'; '#FF0000'; '#00FF00'; '#0000FF'; '#00FFFF'; '#D95319'; '#00FFFF';...

'#0072BD'; '#D95319' ; '#EDB120'; '#7E2F8E'; '#77AC30'; '#4DBEEE'; '#A2142F'; '#FF0000'; '#00FF00'; '#0000FF'; '#00FFFF'; '#D95319'; '#00FFFF'; '#0072BD'; '#D95319' ; '#EDB120'; '#7E2F8E'; '#77AC30'; '#4DBEEE'; '#A2142F'; '#FF0000'; '#00FF00'; '#0000FF'; '#00FFFF'; '#D95319'; '#00FFFF';...

'#0072BD'; '#D95319' ; '#EDB120'; '#7E2F8E'; '#77AC30'; '#4DBEEE'; '#A2142F'; '#FF0000'; '#00FF00'; '#0000FF'; '#00FFFF'; '#D95319'; '#00FFFF';'#0072BD'; '#D95319' ; '#EDB120'; '#7E2F8E'; '#77AC30'; '#4DBEEE'; '#A2142F'; '#FF0000'; '#00FF00'; '#0000FF'; '#00FFFF'; '#D95319'; '#00FFFF';...

'#0072BD'; '#D95319' ; '#EDB120'; '#7E2F8E'; '#77AC30'; '#4DBEEE'; '#A2142F'; '#FF0000'; '#00FF00'; '#0000FF'; '#00FFFF'; '#D95319'; '#00FFFF'; '#0072BD'; '#D95319' ; '#EDB120'; '#7E2F8E'; '#77AC30'; '#4DBEEE'; '#A2142F'; '#FF0000'; '#00FF00'; '#0000FF'; '#00FFFF'; '#D95319'; '#00FFFF';...

'#0072BD'; '#D95319' ; '#EDB120'; '#7E2F8E'; '#77AC30'; '#4DBEEE'; '#A2142F'; '#FF0000'; '#00FF00'; '#0000FF'; '#00FFFF'; '#D95319'; '#00FFFF';'#0072BD'; '#D95319' ; '#EDB120'; '#7E2F8E'; '#77AC30'; '#4DBEEE'; '#A2142F'; '#FF0000'; '#00FF00'; '#0000FF'; '#00FFFF'; '#D95319'; '#00FFFF';...

'#0072BD'; '#D95319' ; '#EDB120'; '#7E2F8E'; '#77AC30'; '#4DBEEE'; '#A2142F'; '#FF0000'; '#00FF00'; '#0000FF'; '#00FFFF'; '#D95319'; '#00FFFF'; '#0072BD'; '#D95319' ; '#EDB120'; '#7E2F8E'; '#77AC30'; '#4DBEEE'; '#A2142F'; '#FF0000'; '#00FF00'; '#0000FF'; '#00FFFF'; '#D95319'; '#00FFFF';...

'#0072BD'; '#D95319' ; '#EDB120'; '#7E2F8E'; '#77AC30'; '#4DBEEE'; '#A2142F'; '#FF0000'; '#00FF00'; '#0000FF'; '#00FFFF'; '#D95319'; '#00FFFF';'#0072BD'; '#D95319' ; '#EDB120'; '#7E2F8E'; '#77AC30'; '#4DBEEE'; '#A2142F'; '#FF0000'; '#00FF00'; '#0000FF'; '#00FFFF'; '#D95319'; '#00FFFF';...

'#0072BD'; '#D95319' ; '#EDB120'; '#7E2F8E'; '#77AC30'; '#4DBEEE'; '#A2142F'; '#FF0000'; '#00FF00'; '#0000FF'; '#00FFFF'; '#D95319'; '#00FFFF'; '#0072BD'; '#D95319' ; '#EDB120'; '#7E2F8E'; '#77AC30'; '#4DBEEE'; '#A2142F'; '#FF0000'; '#00FF00'; '#0000FF'; '#00FFFF'; '#D95319'; '#00FFFF';...

'#0072BD'; '#D95319' ; '#EDB120'; '#7E2F8E'; '#77AC30'; '#4DBEEE'; '#A2142F'; '#FF0000'; '#00FF00'; '#0000FF'; '#00FFFF'; '#D95319'; '#00FFFF'; '#0072BD'; '#D95319' ; '#EDB120'; '#7E2F8E'; '#77AC30'; '#4DBEEE'; '#A2142F'; '#FF0000'; '#00FF00'; '#0000FF'; '#00FFFF'; '#D95319'; '#00FFFF';...

'#0072BD'; '#D95319' ; '#EDB120'; '#7E2F8E'; '#77AC30'; '#4DBEEE'; '#A2142F'; '#FF0000'; '#00FF00'; '#0000FF'; '#00FFFF'; '#D95319'; '#00FFFF'; '#0072BD'; '#D95319' ; '#EDB120'; '#7E2F8E'; '#77AC30'; '#4DBEEE'; '#A2142F'; '#FF0000'; '#00FF00'; '#0000FF'; '#00FFFF'; '#D95319'; '#00FFFF';...

'#0072BD'; '#D95319' ; '#EDB120'; '#7E2F8E'; '#77AC30'; '#4DBEEE'; '#A2142F'; '#FF0000'; '#00FF00'; '#0000FF'; '#00FFFF'; '#D95319'; '#00FFFF';'#0072BD'; '#D95319' ; '#EDB120'; '#7E2F8E'; '#77AC30'; '#4DBEEE'; '#A2142F'; '#FF0000'; '#00FF00'; '#0000FF'; '#00FFFF'; '#D95319'; '#00FFFF';...

'#0072BD'; '#D95319' ; '#EDB120'; '#7E2F8E'; '#77AC30'; '#4DBEEE'; '#A2142F'; '#FF0000'; '#00FF00'; '#0000FF'; '#00FFFF'; '#D95319'; '#00FFFF'; '#0072BD'; '#D95319' ; '#EDB120'; '#7E2F8E'; '#77AC30'; '#4DBEEE'; '#A2142F'; '#FF0000'; '#00FF00'; '#0000FF'; '#00FFFF'; '#D95319'; '#00FFFF';...

'#0072BD'; '#D95319' ; '#EDB120'; '#7E2F8E'; '#77AC30'; '#4DBEEE'; '#A2142F'; '#FF0000'; '#00FF00'; '#0000FF'; '#00FFFF'; '#D95319'; '#00FFFF';'#0072BD'; '#D95319' ; '#EDB120'; '#7E2F8E'; '#77AC30'; '#4DBEEE'; '#A2142F'; '#FF0000'; '#00FF00'; '#0000FF'; '#00FFFF'; '#D95319'; '#00FFFF';...

'#0072BD'; '#D95319' ; '#EDB120'; '#7E2F8E'; '#77AC30'; '#4DBEEE'; '#A2142F'; '#FF0000'; '#00FF00'; '#0000FF'; '#00FFFF'; '#D95319'; '#00FFFF'; '#0072BD'; '#D95319' ; '#EDB120'; '#7E2F8E'; '#77AC30'; '#4DBEEE'; '#A2142F'; '#FF0000'; '#00FF00'; '#0000FF'; '#00FFFF'; '#D95319'; '#00FFFF';...

'#0072BD'; '#D95319' ; '#EDB120'; '#7E2F8E'; '#77AC30'; '#4DBEEE'; '#A2142F'; '#FF0000'; '#00FF00'; '#0000FF'; '#00FFFF'; '#D95319'; '#00FFFF'];

cross_coef = 1.8;

flow = [];

cryostat_neck = [];

consumption_L_h = [];

%here we suppose to have the shield always at 100K and 200K and placed at

%1/3 and 2/3 of the neck length

for i = 1:length(flow_guess)

for j = 1:length(cryostat_neck_guess)

z_span1 = [0 cryostat_neck_guess(j)/3];

F1 = @(z,T) (flow_guess(i)*cp*T - q_He_pot_shield_1*(100^4-4.2^4))/((cross_coef*cross_section*(K_T_304L(1)*T^3+K_T_304L(2)*T^2+K_T_304L(3)*T+K_T_304L(4)))+...

(cross_section_He*((K_T_He(1)*T^3+K_T_He(2)*T^2+K_T_He(3)*T+K_T_He(4)))));

[z1,T_1] = ode45(F1, z_span1, T0);

T2 = T_1(end);

z_span2 = [z1(end) cryostat_neck_guess(j)/3*2];

F2 = @(z,T) (flow_guess(i)*cp*T - q_shield_1_shield_2*(200^4-100^4))/((cross_coef*cross_section*(K_T_304L(1)*T^3+K_T_304L(2)*T^2+K_T_304L(3)*T+K_T_304L(4)))+...

(cross_section_He*((K_T_He(1)*T^3+K_T_He(2)*T^2+K_T_He(3)*T+K_T_He(4)))));

[z2,T_2] = ode45(F2, z_span2, T2);

T3 = T_2(end);

z_span3 = [z2(end) cryostat_neck_guess(j)];

F3 = @(z,T) (flow_guess(i)*cp*T - q_shield_2_OVC*(294^4-200^4))/((cross_coef*cross_section*(K_T_304L(1)*T^3+K_T_304L(2)*T^2+K_T_304L(3)*T+K_T_304L(4)))+...

(cross_section_He*((K_T_He(1)*T^3+K_T_He(2)*T^2+K_T_He(3)*T+K_T_He(4)))));

[z3,T_3] = ode45(F3, z_span3, T3);

if abs(T_3(end) - 294) < 0.5

T_tot = [T_1; T_2; T_3];

z_tot = [z1; z2; z3];

flow = [flow; flow_guess(i)];

consumption_L_h = [consumption_L_h; flow_guess(i)*22.4*3600/750];

cryostat_neck = [cryostat_neck; cryostat_neck_guess(j)];

figure

%plot(z_tot,T_tot,'linewidth', 1, 'color', colors(i,:));

plot(z_tot,T_tot,'linewidth', 1);

hold on

%plot([z1(end) z1(end)], [0 T_1(end)],':','linewidth', 1, 'color', colors(i,:));

%plot([z2(end) z2(end)], [0 T_2(end)],':','linewidth', 1, 'color', colors(i,:));

%plot([0 z1(end)], [T_1(end) T_1(end)],':','linewidth', 1, 'color', colors(i,:));

%plot([0 z2(end)], [T_2(end) T_2(end)],':','linewidth', 1, 'color', colors(i,:));

plot([z1(end) z1(end)], [0 T_1(end)],':','linewidth', 1,'color', '#0072BD');

plot([z2(end) z2(end)], [0 T_2(end)],':','linewidth', 1,'color', '#0072BD');

plot([0 z1(end)], [T_1(end) T_1(end)],':','linewidth', 1,'color', '#0072BD');

plot([0 z2(end)], [T_2(end) T_2(end)],':','linewidth', 1,'color', '#0072BD');

hold off

box on

set(gca,'FontSize',16);

set(gca,'linewidth',2)

xlabel('Temperature (K)');

xlim([0 0.3]);

ylabel('Heat per unit time (W)');

ylim([0 300]);

xlabel('Distance from He pot (m)');

ylabel('Temperature (K)');

legend(append('Neck length = ', num2str(cryostat_neck_guess(j), '%.3f')), 'Location', 'northwest');

text(0.13,15,append('He consumption = ',num2str((flow_guess(i)*22.4*3600/750), '%.3f'), ' L/h'),'FontSize',14)

end

end

end

figure

plot(cryostat_neck, flow, 'o', 'LineWidth', 1.5);

box on

set(gca,'FontSize',16);

set(gca,'linewidth',2)

xlabel('Neck length (m)');

xlim([0.1 0.40]);

ylabel('He gas flow (mol/s)');

ylim([0.002 0.008]);

figure

plot(cryostat_neck, consumption_L_h, 'o', 'LineWidth', 1.5);

hold on

plot([0.19 0.19], [0 0.385],':','linewidth', 1, 'color', '#D95319');

plot([0 0.27], [0.2828 0.2828],':','linewidth', 1, 'color', '#EDB120');

plot([0 0.19], [0.385 0.385],':','linewidth', 1, 'color', '#D95319');

plot([0.27 0.27], [0 0.2828],':','linewidth', 1, 'color', '#EDB120');

box on

set(gca,'FontSize',16);

set(gca,'linewidth',2)

xlabel('Neck length (m)');

xlim([0.1 0.40]);

ylabel('Liquid He consumption (L/h)');

ylim([0.2 0.8]);

legend('solutions', 'max holding time', 'our choice');

He_volume = (0.75^2*pi)*(4-cryostat_neck*10); %dm^3

Holding_time = He_volume./consumption_L_h;

figure

plot(cryostat_neck, Holding_time, 'o', 'LineWidth', 1.5);

hold on

plot([0.19 0.19], [0 9.64],':','linewidth', 1, 'color', '#D95319');

plot([0 0.27], [8.18 8.18],':','linewidth', 1, 'color', '#EDB120');

plot([0 0.19], [9.64 9.64],':','linewidth', 1, 'color', '#D95319');

plot([0.27 0.27], [0 8.18],':','linewidth', 1, 'color', '#EDB120');

box on

set(gca,'FontSize',16);

set(gca,'linewidth',2)

xlabel('Neck length (m)');

xlim([0.1 0.40]);

ylabel('Holding time (h)');

ylim([0 15]);

legend('solutions', 'max holding time', 'our choice');

figure

plot(cryostat_neck, He_volume, 'o', 'LineWidth', 1.5);

hold on

plot([0.19 0.19], [0 3.7],':','linewidth', 1, 'color', '#D95319');

plot([0 0.27], [2.3 2.3],':','linewidth', 1, 'color', '#EDB120');

plot([0 0.19], [3.7 3.7],':','linewidth', 1, 'color', '#D95319');

plot([0.27 0.27], [0 2.3],':','linewidth', 1, 'color', '#EDB120');

box on

set(gca,'FontSize',16);

set(gca,'linewidth',2)

xlabel('Neck length (m)');

xlim([0.1 0.40]);

ylabel('Reservoir volume (L)');

ylim([0 6]);

legend('solutions', 'max holding time', 'our choice');

%%

**5.3 Optimization of shields position**

%This is a thermodynamic model to optimize the position of the shields in a

%small bath cryostat for a given flow rate of the he gas and a given length

%of the neck

%the bath is at 4.2K

%the aim is to find a temperature profile as a function of the distance from the He bath for a given gas flow

%%

clear all

close all

%First of all, we consider only the contribution to the heat transfer of

%the cryostat metal parts: neck and probe (conduction)

%we assume that all is stainless steel

cross_section_neck = pi*((0.0635/2)^2-(0.0625/2)^2);

cross_section_tube = pi*((0.013/2)^2-(0.012/2)^2);

cross_section_square = 0.01^2-0.008^2;

cross_section_syphon = pi*((0.0115/2)^2-(0.0105/2)^2);

cross_section_coax = pi*((0.0032/2)^2-(0.003/2)^2);

cross_section_He_probe = pi*0.001^2;

cross_section = cross_section_He_probe + cross_section_coax + cross_section_neck + cross_section_tube + 4*cross_section_square +...

cross_section_syphon; %m^2

%let's determine the thermal conductivity function of SS304L from experimental

%data. Conductivity is in W*m^-1*K^-1

temp_list_304L = [4, 6, 8, 10, 15, 20, 25, 30, 35, 40, 50, 60, 70, 76, 80,...

90, 100, 120, 140, 160, 180, 200, 250, 300];

cond_list_304L = [0.24, 0.39, 0.57, 0.77, 1.32, 1.95, 2.60, 3.30,...

4.00, 4.70, 5.80, 6.80, 7.60, 8.00, 8.30, 9.00, 9.50, 10.30, 11.00, 12.00, 12.30, 13.00, 14.00, 15.00];

temperature = 4:0.1:300;

cond_304L = interp1(temp_list_304L, cond_list_304L, temperature);

%let's define flow and molar enthalpy: for each z the enthalpy flow is

%flow*H(T) = flow*cp = flow*5/2*R = flow*20.7 J*mol^-1*K^-1

%consideration on the flow: in 1 mole we have 22.4 L; 1 lit of liquid

%helium corresponds to 757 lit of gas; we measure a boil-off rate of 0.3

%L/h ==> 0.0631 lit/s ==> 0.0028 mol/s

cp = 20.7; %J*mol^-1*K^-1

%the equation to solve is flow*cp*T = cross_section*K(T)*dT/dz. For Ode45

%you have to explicit the dT/dz term

%let's get the polynomial expression that approx K_304L(T), we use order 3

K_T_304L = polyfit(temperature, cond_304L, 3);

%Let's solve the differential equation: initial value is T = 4.2 K and the

%we find solution for all the length of the neck (0 ---> 0.27 m)

cryostat_neck = 0.27;

T0 = 4.2;

%lets now introduce the radiation from the shields : Pn(W) =

%emiss_n*Stef_const*Tn^4.

Stef_const = 5.67e-8; %W*m^-2*K^-4

%I write down the emissivity of Al at the

%interesting temperatures

emiss_Al_4K = 0.04;

emiss_Al_100K = 0.08;

emiss_Al_200K = 0.1;

emiss_SS_RT = 0.2;

%I write down all important dimensions of shields, He pot, and OVC. All is

%in meters

He_pot_r = 0.154/2;

He_pot_h = 0.138;

shield_1_r = 0.175/2;

shield_1_h = 0.235;

shield_2_r = 0.185/2;

shield_2_h = 0.340;

OVC_r = 0.197/2;

OVC_h = 0.450;

%I calculate all cylindrical surfaces

He_pot_surf = shield_surface(He_pot_r, He_pot_h);

shield_1_surf = shield_surface(shield_1_r, shield_1_h);

shield_2_surf = shield_surface(shield_2_r, shield_2_h);

OVC_surf = shield_surface(OVC_r, OVC_h);

%I calculate all view factors. The view factor found in the literature

%assumes equal height of the cylinders. We have different values. We put

%the mean to compensate

vf_pot_shiled_1 = view_factor(He_pot_r, shield_1_r, (He_pot_h+shield_1_h)/2);

vf_shiled_1_shiled_2 = view_factor(shield_1_r, shield_2_r, (shield_1_h+shield_2_h)/2);

vf_shiled_2_OVC = view_factor(shield_2_r, OVC_r, (shield_2_h+OVC_h)/2);

%for a pair of screens at temperature T1 and T2 with T1<T1 the power (W) dumped at the

%junction between 1 and the central tube is q_1_2 = Stef_const*(surf1*(T2^4-T1^4)*F12)/(1/eps1+surf1/surf2*(1/eps1-1))

%the function radiation(T1, eps1, S1, T2, eps2, S2, F12) calculates this. I

%do it for the 3 cases of He_pot to shield 1; shield_1 to shield_2;

%shield_2 to OVC

q_He_pot_shield_1 = radiation_2(emiss_Al_4K, He_pot_surf, emiss_Al_100K, shield_1_surf, vf_pot_shiled_1);

q_shield_1_shield_2 = radiation_2(emiss_Al_100K, shield_1_surf, emiss_Al_200K, shield_2_surf, vf_shiled_1_shiled_2);

q_shield_2_OVC = radiation_2(emiss_Al_200K, shield_2_surf, emiss_SS_RT, OVC_surf, vf_shiled_2_OVC);

%now we add the contribution of the heat conduction through the column of gas itself

% from the CRC Handbook we find the thermal conductivity of He gas as a

% fucntion of temperature W*m^-1*K^-1

temp_list_He = [4, 6, 8, 10, 14, 16, 20, 25, 30, 35, 40, 60, 80, 100, 120, 140, 160, 180,...

200, 240, 260, 280, 300];

cond_list_He = 0.001*[9.004, 11.74, 14.49, 16.89, 20.98, 22.81, 26.20, 30.09, 33.72, 37.15,...

40.44, 52.55, 63.52, 73.71, 83.33, 92.5, 101.3, 109.8, 118.0, 133.7, 141.3, 148.7, 156.0];

%we interpolate again

cond_He = interp1(temp_list_He, cond_list_He, temperature);

%let's get the polynomial law that approx K_He(T), we use order 3

K_T_He = polyfit(temperature, cond_He, 3);

%He column cross section is the ID of the neck

cross_section_He = pi*(0.0625/2)^2;

%first of all I find the flow that allows me to reach 100 K after 9 cm

flow_guess = 0.0023:0.00001:0.0028; %mol/s

shield_1_position_guess = 0.070:0.005:0.130;

shield_2_position_guess = 0.170:0.005:0.230;

shield_1_temperature_guess = 70:1:130;

shield_2_temperature_guess = 170:1:230;

colors = ['#0072BD'; '#D95319' ; '#EDB120'; '#7E2F8E'; '#77AC30'; '#4DBEEE'; '#A2142F'; '#FF0000'; '#00FF00'; '#0000FF'; '#00FFFF'; '#D95319'; '#00FFFF';...

'#0072BD'; '#D95319' ; '#EDB120'; '#7E2F8E'; '#77AC30'; '#4DBEEE'; '#A2142F'; '#FF0000'; '#00FF00'; '#0000FF'; '#00FFFF'; '#D95319'; '#00FFFF'; '#0072BD'; '#D95319' ; '#EDB120'; '#7E2F8E'; '#77AC30'; '#4DBEEE'; '#A2142F'; '#FF0000'; '#00FF00'; '#0000FF'; '#00FFFF'; '#D95319'; '#00FFFF';...

'#0072BD'; '#D95319' ; '#EDB120'; '#7E2F8E'; '#77AC30'; '#4DBEEE'; '#A2142F'; '#FF0000'; '#00FF00'; '#0000FF'; '#00FFFF'; '#D95319'; '#00FFFF';'#0072BD'; '#D95319' ; '#EDB120'; '#7E2F8E'; '#77AC30'; '#4DBEEE'; '#A2142F'; '#FF0000'; '#00FF00'; '#0000FF'; '#00FFFF'; '#D95319'; '#00FFFF';...

'#0072BD'; '#D95319' ; '#EDB120'; '#7E2F8E'; '#77AC30'; '#4DBEEE'; '#A2142F'; '#FF0000'; '#00FF00'; '#0000FF'; '#00FFFF'; '#D95319'; '#00FFFF'; '#0072BD'; '#D95319' ; '#EDB120'; '#7E2F8E'; '#77AC30'; '#4DBEEE'; '#A2142F'; '#FF0000'; '#00FF00'; '#0000FF'; '#00FFFF'; '#D95319'; '#00FFFF';...

'#0072BD'; '#D95319' ; '#EDB120'; '#7E2F8E'; '#77AC30'; '#4DBEEE'; '#A2142F'; '#FF0000'; '#00FF00'; '#0000FF'; '#00FFFF'; '#D95319'; '#00FFFF';'#0072BD'; '#D95319' ; '#EDB120'; '#7E2F8E'; '#77AC30'; '#4DBEEE'; '#A2142F'; '#FF0000'; '#00FF00'; '#0000FF'; '#00FFFF'; '#D95319'; '#00FFFF';...

'#0072BD'; '#D95319' ; '#EDB120'; '#7E2F8E'; '#77AC30'; '#4DBEEE'; '#A2142F'; '#FF0000'; '#00FF00'; '#0000FF'; '#00FFFF'; '#D95319'; '#00FFFF'; '#0072BD'; '#D95319' ; '#EDB120'; '#7E2F8E'; '#77AC30'; '#4DBEEE'; '#A2142F'; '#FF0000'; '#00FF00'; '#0000FF'; '#00FFFF'; '#D95319'; '#00FFFF';...

'#0072BD'; '#D95319' ; '#EDB120'; '#7E2F8E'; '#77AC30'; '#4DBEEE'; '#A2142F'; '#FF0000'; '#00FF00'; '#0000FF'; '#00FFFF'; '#D95319'; '#00FFFF'; '#0072BD'; '#D95319' ; '#EDB120'; '#7E2F8E'; '#77AC30'; '#4DBEEE'; '#A2142F'; '#FF0000'; '#00FF00'; '#0000FF'; '#00FFFF'; '#D95319'; '#00FFFF';...

'#0072BD'; '#D95319' ; '#EDB120'; '#7E2F8E'; '#77AC30'; '#4DBEEE'; '#A2142F'; '#FF0000'; '#00FF00'; '#0000FF'; '#00FFFF'; '#D95319'; '#00FFFF'; '#0072BD'; '#D95319' ; '#EDB120'; '#7E2F8E'; '#77AC30'; '#4DBEEE'; '#A2142F'; '#FF0000'; '#00FF00'; '#0000FF'; '#00FFFF'; '#D95319'; '#00FFFF';...

'#0072BD'; '#D95319' ; '#EDB120'; '#7E2F8E'; '#77AC30'; '#4DBEEE'; '#A2142F'; '#FF0000'; '#00FF00'; '#0000FF'; '#00FFFF'; '#D95319'; '#00FFFF';'#0072BD'; '#D95319' ; '#EDB120'; '#7E2F8E'; '#77AC30'; '#4DBEEE'; '#A2142F'; '#FF0000'; '#00FF00'; '#0000FF'; '#00FFFF'; '#D95319'; '#00FFFF';...

'#0072BD'; '#D95319' ; '#EDB120'; '#7E2F8E'; '#77AC30'; '#4DBEEE'; '#A2142F'; '#FF0000'; '#00FF00'; '#0000FF'; '#00FFFF'; '#D95319'; '#00FFFF'; '#0072BD'; '#D95319' ; '#EDB120'; '#7E2F8E'; '#77AC30'; '#4DBEEE'; '#A2142F'; '#FF0000'; '#00FF00'; '#0000FF'; '#00FFFF'; '#D95319'; '#00FFFF';...

'#0072BD'; '#D95319' ; '#EDB120'; '#7E2F8E'; '#77AC30'; '#4DBEEE'; '#A2142F'; '#FF0000'; '#00FF00'; '#0000FF'; '#00FFFF'; '#D95319'; '#00FFFF';'#0072BD'; '#D95319' ; '#EDB120'; '#7E2F8E'; '#77AC30'; '#4DBEEE'; '#A2142F'; '#FF0000'; '#00FF00'; '#0000FF'; '#00FFFF'; '#D95319'; '#00FFFF';...

'#0072BD'; '#D95319' ; '#EDB120'; '#7E2F8E'; '#77AC30'; '#4DBEEE'; '#A2142F'; '#FF0000'; '#00FF00'; '#0000FF'; '#00FFFF'; '#D95319'; '#00FFFF'; '#0072BD'; '#D95319' ; '#EDB120'; '#7E2F8E'; '#77AC30'; '#4DBEEE'; '#A2142F'; '#FF0000'; '#00FF00'; '#0000FF'; '#00FFFF'; '#D95319'; '#00FFFF';...

'#0072BD'; '#D95319' ; '#EDB120'; '#7E2F8E'; '#77AC30'; '#4DBEEE'; '#A2142F'; '#FF0000'; '#00FF00'; '#0000FF'; '#00FFFF'; '#D95319'; '#00FFFF'];

flow =[];

shield_1_position = [];

shield_2_position = [];

shield_1_temperature = [];

shield_2_temperature = [];

consumption_L_h = [];

cross_coef = 1.8;

shields_temperature_ratio = [];

for m = 1:length(shield_1_temperature_guess)

for l = 1:length(shield_2_temperature_guess)

%waitbar(l/length(shield_1_temperature_guess), append('still executing the script: ', num2str(l/length(shield_1_temperature_guess)*100, '%.0f'), '% done'));

for i = 1:length(flow_guess)

for j = 1:length(shield_1_position_guess)

z_span1 = [0 shield_1_position_guess(j)];

F1 = @(z,T) (flow_guess(i)*cp*T - q_He_pot_shield_1*(shield_1_temperature_guess(m)^4-4.2^4))/((cross_coef*cross_section*(K_T_304L(1)*T^3+K_T_304L(2)*T^2+K_T_304L(3)*T+K_T_304L(4)))+...

(cross_section_He*((K_T_He(1)*T^3+K_T_He(2)*T^2+K_T_He(3)*T+K_T_He(4)))));

[z1,T_1] = ode45(F1, z_span1, T0);

T2 = T_1(end);

for k = 1:length(shield_2_position_guess)

z_span2 = [z1(end) shield_2_position_guess(k)];

F2 = @(z,T) (flow_guess(i)*cp*T - q_shield_1_shield_2*(shield_2_temperature_guess(l)^4-shield_1_temperature_guess(m)^4))/((cross_coef*cross_section*(K_T_304L(1)*T^3+K_T_304L(2)*T^2+K_T_304L(3)*T+K_T_304L(4)))+...

(cross_section_He*((K_T_He(1)*T^3+K_T_He(2)*T^2+K_T_He(3)*T+K_T_He(4)))));

[z2,T_2] = ode45(F2, z_span2, T2);

T3 = T_2(end);

z_span3 = [z2(end) 0.27];

F3 = @(z,T) (flow_guess(i)*cp*T - q_shield_2_OVC*(294^4-shield_2_temperature_guess(l)^4))/((cross_coef*cross_section*(K_T_304L(1)*T^3+K_T_304L(2)*T^2+K_T_304L(3)*T+K_T_304L(4)))+...

(cross_section_He*((K_T_He(1)*T^3+K_T_He(2)*T^2+K_T_He(3)*T+K_T_He(4)))));

[z3,T_3] = ode45(F3, z_span3, T3);

if abs(T_2(end) - shield_2_temperature_guess(l)) < 0.5 && abs(T_1(end) - shield_1_temperature_guess(m)) < 0.5 && abs(T_3(end) - 294) < 0.5

T_tot = [T_1; T_2; T_3];

z_tot = [z1; z2; z3];

shield_2_position = [shield_2_position; shield_2_position_guess(k)];

shield_1_position = [shield_1_position; shield_1_position_guess(j)];

flow = [flow; flow_guess(i)];

consumption_L_h = [consumption_L_h; flow_guess(i)*22.4*3600/750];

shield_1_temperature = [shield_1_temperature; shield_1_temperature_guess(m)];

shield_2_temperature = [shield_2_temperature; shield_2_temperature_guess(l)];

shields_temperature_ratio = [shields_temperature_ratio; shield_2_temperature_guess(l)/shield_1_temperature_guess(m)];

figure

plot(z_tot,T_tot,'linewidth', 1, 'color', colors(l,:));

hold on

plot([z1(end) z1(end)], [0 T_1(end)],':','linewidth', 1, 'color', colors(l,:));

plot([z2(end) z2(end)], [0 T_2(end)],':','linewidth', 1, 'color', colors(l,:));

plot([0 z1(end)], [T_1(end) T_1(end)],':','linewidth', 1, 'color', colors(l,:));

plot([0 z2(end)], [T_2(end) T_2(end)],':','linewidth', 1, 'color', colors(l,:));

hold off

box on

set(gca,'FontSize',16);

set(gca,'linewidth',2)

xlabel('Temperature (K)');

xlim([0 0.3]);

ylabel('Heat per unit time (W)');

ylim([0 300]);

xlabel('Distance from He pot (m)');

ylabel('Temperature (K)');

legend(append('S1 T = ', num2str((shield_1_temperature_guess(m)), '%.0f'), ' K', ', S2 T = ', num2str((shield_2_temperature_guess(l)), '%.0f'), ' K'), 'Location', 'northwest');

text(0.13,15,append('He consumption = ',num2str((flow_guess(i)*22.4*3600/750), '%.3f'), ' L/h'),'FontSize',14)

end

end

end

end

end

end

figure

hold on

plot(shield_1_position, consumption_L_h, 'o', 'LineWidth', 1.5);

plot(shield_2_position, consumption_L_h, '*', 'LineWidth', 1.5);

for p = 1:length(consumption_L_h)

if abs(consumption_L_h(p) - 0.2839) < 0.0001

plot([shield_1_position(p) shield_2_position(p)], [consumption_L_h(p) consumption_L_h(p)],':','linewidth', 1, 'color', [1 0 0]);

elseif consumption_L_h(p) == min (consumption_L_h)

plot([shield_1_position(p) shield_2_position(p)], [consumption_L_h(p) consumption_L_h(p)],':','linewidth', 1, 'color', [0 0 1]);

else

plot([shield_1_position(p) shield_2_position(p)], [consumption_L_h(p) consumption_L_h(p)],':','linewidth', 1, 'color', [0.7 0.7 0.7]);

end

end

box on

set(gca,'FontSize',16);

set(gca,'linewidth',2)

xlabel('Distance from He pot (m)');

xlim([0.05 0.25]);

ylabel('Liquid He consumption (L/h)');

ylim([0.27 0.29]);

legend('Shield 1', 'Shield 2');

figure

plot(shields_temperature_ratio, flow, 'o','LineWidth', 1.5);

box on

set(gca,'FontSize',16);

set(gca,'linewidth',2)

xlabel('Shields temperature ratio');

xlim([1 4]);

ylabel('He gas flow (mol/s)');

ylim([0.0024 0.0028]);

figure

plot(shields_temperature_ratio, consumption_L_h, 'o','LineWidth', 1.5);

box on

set(gca,'FontSize',16);

set(gca,'linewidth',2)

xlabel('Shields temperature ratio');

xlim([1 4]);

ylabel('Liquid He consumption (L/h)');

ylim([0.27 0.29]);

figure

plot(shield_2_position-shield_1_position , consumption_L_h, 'o','LineWidth', 1.5);

box on

set(gca,'FontSize',16);

set(gca,'linewidth',2)

xlabel('Shields distance (m)');

xlim([0 0.2]);

ylabel('Liquid He consumption (L/h)');

ylim([0.27 0.29]);

p1 = polyfit(shield_1_position, shield_1_temperature, 1);

p2 = polyfit(shield_2_position, shield_2_temperature, 1);

f1 = polyval(p1, shield_1_position);

f2 = polyval(p2, shield_2_position);

figure

hold on

plot(shield_1_position, shield_1_temperature, 'o', 'LineWidth', 1.5);

plot(shield_2_position, shield_2_temperature, '*', 'LineWidth', 1.5);

plot(shield_1_position , f1, ':','LineWidth', 1.5, 'color', '#D95319');

plot(shield_2_position , f2, ':','LineWidth', 1.5, 'color', '#D95319');

box on

set(gca,'FontSize',16);

set(gca,'linewidth',2)

xlabel('Distance from He pot (m)');

xlim([0.05 0.25]);

ylabel('Temperature (K)');

ylim([50 250]);

legend('Shield 1', 'Shield 2', 'Linear fit', 'Location', 'southeast');

%%

**5.3 Optimization of shields position**

%This is a thermodynamic model to optimize the position of the shield in a

%small bath cryostat for a given flow rate of the he gas

%the bath is at 4.2K

%the aim is to find a temperature profile as a function of the distance from the He bath for a given gas flow

%%

clear all

close all

%First of all, we con

%sider only the contribution to the heat transfer of

%the cryostat metal parts: neck and probe (conduction)

%we assume that all is stainless steel

cross_section_neck = pi*((0.0635/2)^2-(0.0625/2)^2);

cross_section_tube = pi*((0.013/2)^2-(0.012/2)^2);

cross_section_square = 0.01^2-0.008^2;

cross_section_syphon = pi*((0.0115/2)^2-(0.0105/2)^2);

cross_section_coax = pi*((0.0032/2)^2-(0.003/2)^2);

cross_section_He_probe = pi*0.002^2;

cross_section = cross_section_He_probe + cross_section_coax + cross_section_neck + cross_section_tube + 4*cross_section_square +...

cross_section_syphon; %m^2

%let's determine the thermal conducitivy function of SS304L from experimetal

%data. Conductivity is in W*m^-1*K^-1

temp_list_304L = [4, 6, 8, 10, 15, 20, 25, 30, 35, 40, 50, 60, 70, 76, 80,...

90, 100, 120, 140, 160, 180, 200, 250, 300];

cond_list_304L = [0.24, 0.39, 0.57, 0.77, 1.32, 1.95, 2.60, 3.30,...

4.00, 4.70, 5.80, 6.80, 7.60, 8.00, 8.30, 9.00, 9.50, 10.30, 11.00, 12.00, 12.30, 13.00, 14.00, 15.00];

temperature = 4:0.1:300;

cond_304L = interp1(temp_list_304L, cond_list_304L, temperature);

%let's define flow and molar entalpy: for each z the enthalpy flow is

%flow*H(T) = flow*cp = flow*5/2*R = flow*20.7 J*mol^-1*K^-1

%consideration on the flow: in 1 mole we have 22.4 L; 1 lit of liquid

%helium corresponds to 757 lit of gas; we measure a boil-off rate of 0.3

%L/h ==> 0.0631 lit/s ==> 0.0028 mol/s

cp = 20.7; %J*mol^-1*K^-1

%the equation to solve is flow*cp*T = cross_section*K(T)*dT/dz. For Ode45

%you have to explicit the dT/dz term

%let's get the polynomial expresion that approx K_304L(T), we use order 3

K_T_304L = polyfit(temperature, cond_304L, 3);

%Let's solve the differential equation: initial value is T = 4.2 K and the

%we find solution for all the length of the neck (0 ---> 0.27 m)

cryostat_neck = 0.20;

T0 = 4.2;

%lets now introduce the radiation from the shields : Pn(W) =

%emiss_n*Stef_const*Tn^4.

Stef_const = 5.67e-8; %W*m^-2*K^-4

%I write down the emissivity of Al at the

%interesting temperatures

emiss_Al_4K = 0.04;

emiss_Al_100K = 0.08;

emiss_Al_200K = 0.1;

emiss_SS_RT = 0.2;

%I write down all important dimensions of shields, He pot, and OVC. All is

%in meters

He_pot_r = 0.154/2;

He_pot_h = 0.138;

shield_1_r = 0.175/2;

shield_1_h = 0.235;

shield_2_r = 0.185/2;

shield_2_h = 0.340;

OVC_r = 0.197/2;

OVC_h = 0.450;

%I calculate all cylindrical surfaces

He_pot_surf = shield_surface(He_pot_r, He_pot_h);

shield_1_surf = shield_surface(shield_1_r, shield_1_h);

shield_2_surf = shield_surface(shield_2_r, shield_2_h);

OVC_surf = shield_surface(OVC_r, OVC_h);

%I calculate all view factors. The view factor found in the literature

%assumes equal height of the cylinders. We have different values. We put

%the mean to compensate

vf_pot_shiled_1 = view_factor(He_pot_r, shield_1_r, (He_pot_h+shield_1_h)/2);

vf_shiled_1_OVC = view_factor(shield_1_r, OVC_r, (shield_1_h+OVC_h)/2);

%for a pair of screens at temperature T1 and T2 with T1<T1 the power (W) dumped at the

%junction between 1 and the central tube is q_1_2 = Stef_const*(surf1*(T2^4-T1^4)*F12)/(1/eps1+surf1/surf2*(1/eps1-1))

%the function radiation(T1, eps1, S1, T2, eps2, S2, F12) calculates this. I

%do it for the 3 cases of He_pot to shield 1; shield_1 to shield_2;

%shield_2 to OVC

q_He_pot_shield_1 = radiation_2(emiss_Al_4K, He_pot_surf, emiss_Al_100K, shield_1_surf, vf_pot_shiled_1);

%q_shield_1_shield_2 = radiation_2(emiss_Al_100K, shield_1_surf, emiss_Al_200K, shield_2_surf, vf_shiled_1_shiled_2);

q_shield_1_OVC = radiation_2(emiss_Al_100K, shield_1_surf, emiss_SS_RT, OVC_surf, vf_shiled_1_OVC);

%now we add the contribution of the heat conduction through the column of gas itself

% from the CRC Handbook we find the thermal conductivity of He gas as a

% fucntion of temperature W*m^-1*K^-1

temp_list_He = [4, 6, 8, 10, 14, 16, 20, 25, 30, 35, 40, 60, 80, 100, 120, 140, 160, 180,...

200, 240, 260, 280, 300];

cond_list_He = 0.001*[9.004, 11.74, 14.49, 16.89, 20.98, 22.81, 26.20, 30.09, 33.72, 37.15,...

40.44, 52.55, 63.52, 73.71, 83.33, 92.5, 101.3, 109.8, 118.0, 133.7, 141.3, 148.7, 156.0];

%we interpolate again

cond_He = interp1(temp_list_He, cond_list_He, temperature);

%let's get the polynomial law that approx K_He(T), we use order 3

K_T_He = polyfit(temperature, cond_He, 3);

%He column cross section is the ID of the neck

cross_section_He = pi*0.03^2;

%first of all I find the flow that allows me to reach 100 K after 9 cm

flow_guess = 0.003:0.00001:0.005; %mol/s

shield_1_position_guess = 0.05:0.005:0.16;

shield_1_temperature_guess = 70:1:216;

colors = ['#0072BD'; '#D95319' ; '#EDB120'; '#7E2F8E'; '#77AC30'; '#4DBEEE'; '#A2142F'; '#FF0000'; '#00FF00'; '#0000FF'; '#00FFFF'; '#D95319'; '#00FFFF';...

'#0072BD'; '#D95319' ; '#EDB120'; '#7E2F8E'; '#77AC30'; '#4DBEEE'; '#A2142F'; '#FF0000'; '#00FF00'; '#0000FF'; '#00FFFF'; '#D95319'; '#00FFFF'; '#0072BD'; '#D95319' ; '#EDB120'; '#7E2F8E'; '#77AC30'; '#4DBEEE'; '#A2142F'; '#FF0000'; '#00FF00'; '#0000FF'; '#00FFFF'; '#D95319'; '#00FFFF';...

'#0072BD'; '#D95319' ; '#EDB120'; '#7E2F8E'; '#77AC30'; '#4DBEEE'; '#A2142F'; '#FF0000'; '#00FF00'; '#0000FF'; '#00FFFF'; '#D95319'; '#00FFFF'; '#0072BD'; '#D95319' ; '#EDB120'; '#7E2F8E'; '#77AC30'; '#4DBEEE'; '#A2142F'; '#FF0000'; '#00FF00'; '#0000FF'; '#00FFFF'; '#D95319'; '#00FFFF';...

'#0072BD'; '#D95319' ; '#EDB120'; '#7E2F8E'; '#77AC30'; '#4DBEEE'; '#A2142F'; '#FF0000'; '#00FF00'; '#0000FF'; '#00FFFF'; '#D95319'; '#00FFFF'; '#0072BD'; '#D95319' ; '#EDB120'; '#7E2F8E'; '#77AC30'; '#4DBEEE'; '#A2142F'; '#FF0000'; '#00FF00'; '#0000FF'; '#00FFFF'; '#D95319'; '#00FFFF';...

'#0072BD'; '#D95319' ; '#EDB120'; '#7E2F8E'; '#77AC30'; '#4DBEEE'; '#A2142F'; '#FF0000'; '#00FF00'; '#0000FF'; '#00FFFF'; '#D95319'; '#00FFFF'; '#0072BD'; '#D95319' ; '#EDB120'; '#7E2F8E'; '#77AC30'; '#4DBEEE'; '#A2142F'; '#FF0000'; '#00FF00'; '#0000FF'; '#00FFFF'; '#D95319'; '#00FFFF';...

'#0072BD'; '#D95319' ; '#EDB120'; '#7E2F8E'; '#77AC30'; '#4DBEEE'; '#A2142F'; '#FF0000'; '#00FF00'; '#0000FF'; '#00FFFF'; '#D95319'; '#00FFFF'; '#0072BD'; '#D95319' ; '#EDB120'; '#7E2F8E'; '#77AC30'; '#4DBEEE'; '#A2142F'; '#FF0000'; '#00FF00'; '#0000FF'; '#00FFFF'; '#D95319'; '#00FFFF';...

'#0072BD'; '#D95319' ; '#EDB120'; '#7E2F8E'; '#77AC30'; '#4DBEEE'; '#A2142F'; '#FF0000'; '#00FF00'; '#0000FF'; '#00FFFF'; '#D95319'; '#00FFFF'; '#0072BD'; '#D95319' ; '#EDB120'; '#7E2F8E'; '#77AC30'; '#4DBEEE'; '#A2142F'; '#FF0000'; '#00FF00'; '#0000FF'; '#00FFFF'; '#D95319'; '#00FFFF';...

'#0072BD'; '#D95319' ; '#EDB120'; '#7E2F8E'; '#77AC30'; '#4DBEEE'; '#A2142F'; '#FF0000'; '#00FF00'; '#0000FF'; '#00FFFF'; '#D95319'; '#00FFFF'; '#0072BD'; '#D95319' ; '#EDB120'; '#7E2F8E'; '#77AC30'; '#4DBEEE'; '#A2142F'; '#FF0000'; '#00FF00'; '#0000FF'; '#00FFFF'; '#D95319'; '#00FFFF';...

'#0072BD'; '#D95319' ; '#EDB120'; '#7E2F8E'; '#77AC30'; '#4DBEEE'; '#A2142F'; '#FF0000'; '#00FF00'; '#0000FF'; '#00FFFF'; '#D95319'; '#00FFFF'];

flow =[];

shield_1_position = [];

shield_temperature = [];

consumption_L_h = [];

for m = 1:length(shield_1_temperature_guess)

for i = 1:length(flow_guess)

for j = 1:length(shield_1_position_guess)

z_span1 = [0 shield_1_position_guess(j)];

F1 = @(z,T) (flow_guess(i)*cp*T - q_He_pot_shield_1*(shield_1_temperature_guess(m)^4-4.2^4))/((1.8*cross_section*(K_T_304L(1)*T^3+K_T_304L(2)*T^2+K_T_304L(3)*T+K_T_304L(4)))+...

(cross_section_He*((K_T_He(1)*T^3+K_T_He(2)*T^2+K_T_He(3)*T+K_T_He(4)))));

[z1,T_1] = ode45(F1, z_span1, T0);

T2 = T_1(end);

z_span2 = [z1(end) 0.20];

F2 = @(z,T) (flow_guess(i)*cp*T - q_shield_1_OVC*(294^4-shield_1_temperature_guess(m)^4))/((1.8*cross_section*(K_T_304L(1)*T^3+K_T_304L(2)*T^2+K_T_304L(3)*T+K_T_304L(4)))+...

(cross_section_He*((K_T_He(1)*T^3+K_T_He(2)*T^2+K_T_He(3)*T+K_T_He(4)))));

[z2,T_2] = ode45(F2, z_span2, T2);

if abs(T_2(end) - 294) < 0.5 && abs(T_1(end) - shield_1_temperature_guess(m)) < 0.5

T_tot = [T_1; T_2];

z_tot = [z1; z2];

shield_1_position = [shield_1_position; shield_1_position_guess(j)];

shield_temperature = [shield_temperature; T_1(end)];

flow = [flow; flow_guess(i)];

consumption_L_h = [consumption_L_h; flow_guess(i)*22.4*3600/750];

figure

plot(z_tot,T_tot,'linewidth', 1, 'color', colors(m,:));

hold on

plot([z1(end) z1(end)], [0 T_1(end)],':','linewidth', 1, 'color', colors(m,:));

plot([0 z1(end)], [T_1(end) T_1(end)],':','linewidth', 1, 'color', colors(m,:));

hold off

box on

set(gca,'FontSize',16);

set(gca,'linewidth',2)

xlabel('Temperature (K)');

xlim([0 0.2]);

ylabel('Heat per unit time (W)');

ylim([0 300]);

xlabel('Distance from He pot (m)');

ylabel('Temperature (K)');

legend(append('Shield Temp = ', num2str((shield_1_temperature_guess(m)), '%.3f')), 'Location', 'northwest');

text(0.06,15,append('He consumption = ',num2str((flow_guess(i)*22.4*3600/750), '%.3f'), ' L/h'),'FontSize',14)

end

end

end

end

figure

hold on

plot(shield_1_position, flow, 'o', 'LineWidth', 1.5);

box on

set(gca,'FontSize',16);

set(gca,'linewidth',2)

xlabel('Distance from He pot (m)');

xlim([0.04 0.17]);

ylabel('He gas flow (mol/s)');

ylim([0.0032 0.004]);

figure

plot(shield_temperature, flow, 'o','LineWidth', 1.5);

box on

set(gca,'FontSize',16);

set(gca,'linewidth',2)

xlabel('Shield temperature (K)');

xlim([60 220]);

ylabel('He gas flow (mol/s)');

ylim([0.0032 0.004]);

min_consumption = find(consumption_L_h == min(consumption_L_h));

figure

plot(shield_temperature, consumption_L_h, 'o','LineWidth', 1.5);

hold on

box on

set(gca,'FontSize',16);

set(gca,'linewidth',2)

xlabel('Shield temperature (K)');

xlim([60 220]);

ylabel('Liquid He consumption (L/h)');

ylim([0.35 0.42]);

plot([shield_temperature(14) shield_temperature(14)], [0 consumption_L_h(14)],':','linewidth', 1, 'color', '#D95319');

plot([0 shield_temperature(14)], [consumption_L_h(14) consumption_L_h(14)],':','linewidth', 1, 'color', '#D95319');

legend('solutions', 'best value');

%plot([0.19 0.19], [0 3.7],':','linewidth', 1, 'color', '#D95319');

%plot([0 0.19], [3.7 3.7],':','linewidth', 1, 'color', '#D95319');

figure

plot(shield_1_position , consumption_L_h, 'o','LineWidth', 1.5);

hold on

box on

set(gca,'FontSize',16);

set(gca,'linewidth',2)

xlabel('Distance from He pot (m)');

xlim([0.04 0.20]);

ylabel('Liquid He consumption (L/h)');

ylim([0.35 0.42]);

plot([shield_1_position(14) shield_1_position(14)], [0 consumption_L_h(14)],':','linewidth', 1, 'color', '#D95319');

plot([0 shield_1_position(14)], [consumption_L_h(14) consumption_L_h(14)],':','linewidth', 1, 'color', '#D95319');

legend('solutions', 'best value');

He_volume = 1*(0.75^2*pi); %dm^3

Holding_time = He_volume./consumption_L_h;

max_holding_time = find(Holding_time == min(Holding_time));

figure

plot(shield_1_position, Holding_time, 'o', 'LineWidth', 1.5);

hold on

box on

set(gca,'FontSize',16);

set(gca,'linewidth',2)

xlabel('Distance from He pot (m)');

xlim([0.04 0.20]);

ylabel('Holding time (h)');

ylim([4 5]);

plot([shield_1_position(14) shield_1_position(14)], [0 Holding_time(14)],':','linewidth', 1, 'color', '#D95319');

plot([0 shield_1_position(14)], [Holding_time(14) Holding_time(14)],':','linewidth', 1, 'color', '#D95319');

legend('solutions', 'best value');

figure

plot(shield_1_position , shield_temperature, 'o','LineWidth', 1.5);

hold on

p = polyfit(shield_1_position, shield_temperature, 1);

f1 = polyval(p, shield_1_position);

plot(shield_1_position , f1, ':','LineWidth', 1.5, 'color', '#D95319');

box on

set(gca,'FontSize',16);

set(gca,'linewidth',2);

xlabel('Distance from He pot (m)');

xlim([0.04 0.20]);

ylabel('Shield temperature (K)');

ylim([60 220]);

legend('solutions', 'linear fit');

%%

**References**

1. Parma, V. Cryostat Design. (2014) doi:10.5170/CERN-2014-005.353.

2. Lide, D. R. *CRC Handbook of Chemistry and Physics*. (CRC Press /Taylor and Francis, Boca Raton, 2009).

3. Duthil, P. Material Properties at Low Temperature. (2014) doi:10.5170/CERN-2014-005.77.
